# Automated urinalysis for predicting urine culture positivity and presumptive Gram classification: a retrospective study at a cancer hospital

**DOI:** 10.3389/fcimb.2026.1935652

**Published:** 2026-10-12

**Authors:** Xinjian Cai, Xijun Liu, Qiuxia Lu, Yiteng Lin

**Affiliations:** Department of Clinical Laboratory Medicine, National Cancer Center/National Clinical Research Center for Cancer/Cancer Hospital & Shenzhen Hospital, Chinese Academy of Medical Sciences and Peking Union Medical College, Shenzhen, China

**Keywords:** automated urinalysis, bacterial particle count, culture-ordering selection, presumptive Gram classification, urine culture positivity

## Abstract

**Introduction:**

Automated urinalysis may help prioritize urine cultures, but whether multivariable models improve on bacterial count alone remains uncertain. We assessed their comparative performance and evaluated presumptive Gram classification in a cancer-hospital cohort.

**Methods:**

We retrospectively analyzed 621 urine specimens from 477 patients with paired urinalysis and clinically requested cultures at a cancer hospital (June 2023–June 2024). Urinalysis measurements were obtained using the Sysmex UF-4000 and UC-3500. Culture outcomes were determined from final laboratory reports; 136 specimens classified as likely contaminated were excluded from the primary analysis. LASSO, random forest, and gradient boosting were compared with bacterial count alone using repeated, nested cross-validation grouped by patient. An exploratory temporal evaluation included later specimens from patients absent from the training cohort.

**Results:**

The primary analysis included 485 specimens, of which 236 were culture-positive. Mean pooled cross-validated area under the receiver operating characteristic curve (AUC) was 0.892 for bacterial count. Corresponding AUCs were 0.895 for LASSO, 0.897 for random forest, and 0.893 for gradient boosting. Patient-cluster 95% confidence intervals for AUC differences versus bacterial count included zero. In temporal evaluation involving 114 specimens from 101 patients, AUCs were 0.927 (95% CI 0.877–0.965) for bacterial count and 0.919 (95% CI 0.860–0.963) for LASSO. Among 211 culture-positive specimens yielding a single bacterial organism, 153 (72.5%) had definite Gram classifications. Accuracy was 94.1% in this subset, compared with 85.6% for an always-Gram-negative prediction. Correct definite classifications were obtained for 68.2% of all 211 specimens.

**Discussion:**

Bacterial count alone provided good discrimination of urine culture positivity in this cancer-hospital cohort, with no demonstrated improvement from multivariable models. Presumptive Gram classification added microbiological information for specimens with definite flags. These findings support prospective evaluation of bacterial count for urine culture triage, with Gram classification as an adjunct.

## 1 Introduction

Urinary tract infection (UTI) is a common reason for urinalysis and urine culture in ambulatory and hospital settings (Flores-Mireles et al., 2015; Foxman, 2014). Culture detects microbial growth and enables organism identification and, when indicated, antimicrobial susceptibility testing. A positive culture alone does not establish symptomatic UTI or the need for antimicrobial treatment; interpretation requires clinical information (Nicolle et al., 2019). Culture commonly requires 24–48 h, and specimens submitted for suspected UTI may show no growth or mixed growth (Bekeris et al., 2008; Broeren et al., 2011). Rapid urinalysis can therefore be evaluated against a microbiological laboratory endpoint, while its effects on treatment and patient outcomes require separate study.

Automated urine particle analyzers, including the Sysmex UF series, and urine chemistry analyzers provide bacterial and leukocyte counts, other particle measurements, dipstick markers, and analyzer-derived flags. Previous studies have evaluated these measurements for predicting culture results and presumptive Gram classification (Broeren et al., 2011; De Rosa et al., 2018; Kim et al., 2018; Wang et al., 2023), while related work has assessed dipstick tests, culture-screening rules, and performance in different populations (Devillé et al., 2004; Jolkkonen et al., 2010; Liu et al., 2024; Chang et al., 2026). Machine-learning approaches and changes to reflex-culture policies have also been investigated (Taylor et al., 2018; Burton et al., 2019; Claeys et al., 2021; Penney et al., 2023; Berger et al., 2025). These studies motivate comparison with simple baselines, but differences in clinical context, outcome definitions, and implementation mean that performance and benefits cannot be assumed to transfer to specimens already selected for culture at a cancer hospital.

Two questions motivate the present laboratory study. First, does multivariable modeling add useful discriminatory information beyond the bacterial particle count in specimens already selected for culture? Second, how does that selection affect the interpretation of predictive values and retrospective decision analyses? Calibration and decision-curve analysis complement discrimination, but neither establishes clinical benefit without a defensible decision context (Van Calster et al., 2019; Vickers and Elkin, 2006). Specimens submitted for culture may differ in bacterial burden and other measured characteristics from the overall population of specimens undergoing urinalysis. Comparing cultured and non-cultured specimens can help characterize these differences. However, without culture results for the non-cultured specimens, we cannot determine their culture positivity rate or assess how a screening strategy would perform in that group. Analyzer-derived Gram classification should also be compared with a simple baseline: assigning every specimen to the most common Gram category in the evaluation set. In addition, the proportion of specimens for which the analyzer provides an interpretable Gram classification should be reported.

We evaluated automated urinalysis for predicting report-defined urine culture positivity in a tertiary cancer-hospital cohort already selected for culture. We compared LASSO-penalized logistic regression and machine-learning models with simple urinalysis comparators, characterized differences between cultured and non-cultured specimens, and assessed internal validation, calibration, and retrospective decision scenarios. We also evaluated presumptive Gram classification against culture and a majority-class baseline. Prediction of rule-defined likely contamination was an exploratory secondary analysis.

## 2 Materials and methods

### 2.1 Study design and data source

This single-center retrospective study used routine laboratory records from a tertiary cancer hospital between 1 June 2023 and 27 June 2024. Automated urinalysis was performed using the UC-3500 urine chemistry analyzer and UF-4000 urine particle analyzer (Sysmex Corporation, Kobe, Japan). After exclusion of quality-control, instrument-error, masked and other non-clinical placeholder records and deduplication by specimen number, the reference workflow comprised 38,307 unique clinical urine specimens. Urinalysis and culture records were linked by specimen number: 643 specimens had a paired culture, of which 621 had an available UF bacterial count and formed the analysis cohort. The 22 specimens without an available UF bacterial count were excluded; absence of an available value does not establish that UF analysis was not performed. The 37,664 specimens without paired culture were used only to describe culture-ordering selection, not as culture-negative controls or as a validation cohort. Details of data cleaning and record correction are provided in the **Supplementary Methods**. Patient identifiers were available for grouping repeated specimens, but symptoms, antimicrobial exposure, neutropenia, detailed oncological status, and patient outcomes were unavailable for clinical adjudication.

### 2.2 Culture reports and study outcome definitions

Clean-catch midstream urine specimens were processed according to the laboratory’s routine procedures. The transport, quantitative inoculation and incubation procedures described below are consistent with the corresponding provisions of WS/T 489–2024, cited here as a retrospective reference because it took effect after the study period (National Health Commission of the People's Republic of China, 2024). The handling policy was delivery within 2 h at room temperature; if delivery was delayed, refrigeration at 2–8 °C or a preservative-containing transport tube was used, with storage/transport not exceeding 24 h. Specimens were inoculated promptly on receipt. After gentle mixing, a 10-μL urine aliquot was used for quantitative inoculation on blood agar; a 1-μL loop was used for subsequent sectional streaking and did not define the quantitative inoculum. Counts on the quantitative plate were multiplied by 100 to obtain CFU/mL. MacConkey and chocolate agar were used for organism isolation. Routine aerobic incubation was at 35–37 °C, with examination at 18–24 h. Under the reading policy, this was the routine final-reading interval unless prolonged incubation was indicated; cultures were extended to 48 h for invasive specimens, tiny or sparse colonies, discordance with Gram-stain findings or clinical symptoms, or immunocompromised patients. This policy does not imply that all specimens in this cancer-hospital cohort underwent 48-h incubation. Isolate identification used a bioMérieux VITEK matrix-assisted laser desorption/ionization time-of-flight mass spectrometry (MALDI-TOF MS) system. The laboratory reported growth below 10³ CFU/mL as <10³ CFU/mL regardless of the number of organisms and could recommend recollection. Identification-only reports could be issued for single-organism growth from 10³ to <10^4^ CFU/mL. For two-organism growth, identification and susceptibility testing could differ between the dominant high-count organism and a lower-count second organism. Mixed growth of at least three organisms or otherwise poor-quality growth prompted assessment and possible recollection.

Urine culture results were classified as negative, positive, or likely contaminated based on the final laboratory reports and the organisms recovered. For clean-catch midstream urine yielding a single pathogen, the laboratory’s reporting thresholds during the study period for diagnostically significant growth were ≥10^5^ CFU/mL for Gram-negative bacteria and ≥10^4^ CFU/mL for Gram-positive bacteria. Among the 621 specimens with available UF bacterial counts, 249 were classified as negative, including 146 with no growth and 103 with counts below 10³ CFU/mL. The 236 positive specimens included cultures yielding one or two organisms and those yielding yeast alone. Of the 136 specimens classified as likely contaminated, 116 were reported as suspected contamination, including 115 with growth of three or more microorganisms. The other 20 yielded single organisms commonly associated with skin or urogenital microbiota: *Staphylococcus epidermidis* (n = 7), *S. lugdunensis* (n = 1), *Corynebacterium* spp. (n = 7), *Lactobacillus iners* (n = 1), *Lactobacillus acidophilus* (n = 1), *Ligilactobacillus salivarius* (n = 1), *Gardnerella vaginalis* (n = 1), and *Streptococcus oralis* (n = 1). These were categorized as likely contaminants for the analysis. Bacterial cultures classified as positive met the applicable laboratory thresholds. The 19 two-organism positive cultures yielded two recognized urinary pathogens, each meeting its applicable quantitative threshold. Yeast growth was identified and reported as culture-positive under the laboratory reporting policy. These categories describe laboratory growth, not clinically adjudicated bacterial co-infection or fungal UTI. Because bacterial particle count does not directly quantify yeast growth, separate sensitivity analyses excluded yeast-only and two-organism cultures (**Supplementary Table 6**).

### 2.3 Predictors and assessment of presumptive Gram classification

The primary analysis predicted urine culture positivity among 485 specimens after excluding those classified as likely contaminated based on culture results. An exploratory secondary analysis assessed the prediction of likely contamination among all 621 specimens.

The culture-positivity models included 18 candidate predictors: bacterial, white blood cell, red blood cell, and epithelial cell counts; conductivity; nitrite; leukocyte esterase; blood; protein; pH; specific gravity; turbidity; white blood cell clumps; the analyzer-generated UTI flag; presumptive Gram-negative and Gram-positive flags; age; and sex. Cell and bacterial counts and conductivity were transformed using ln(1 + x). Bacterial counts reported as >100,000 particles/μL in 53 specimens were entered as 100,000 particles/μL. Red blood cell counts were missing in two specimens and were imputed using the training-set median. Text-derived flags were coded as zero when the corresponding descriptor was absent. For BACT Info, the explicit report categories of no alert and unclassified were both coded as zero for the Gram-negative and Gram-positive indicator variables; these codes do not denote culture negativity or missing data. Blank descriptors in other text-derived fields remain subject to the recording limitations described in the **Supplementary Methods**. Imputation and standardization, where required, were fitted within the training partitions. Predictor coding and the 11 variables used in the contamination model are detailed in the **Supplementary Methods**. The distribution of organisms was summarized for culture-positive specimens. Presumptive Gram classification was evaluated in specimens yielding a single bacterial organism with an identifiable Gram category. Analyzer results were classified as definite when only one Gram flag was recorded, ambiguous when both flags were recorded, and either unclassified or without a BACT Info flag according to the original report descriptor when neither Gram flag was present. Definite-result coverage was calculated by dividing the number of specimens with only a Gram-negative or only a Gram-positive flag by the total number of specimens included in the Gram classification analysis. Among specimens with definite results, performance was assessed using accuracy, sensitivity and specificity for Gram-negative identification, balanced accuracy, and Cohen’s κ, with an always-Gram-negative prediction used as the comparator. We also calculated the percentage of all eligible specimens with a correct, definite Gram classification. Specimens with dual flags, unclassified results or no BACT Info flag remained in the denominators for coverage and this overall percentage.

### 2.4 Statistical analysis

#### 2.4.1 Model development and comparators

LASSO-penalized logistic regression was the primary fitted model (Tibshirani, 1996; Friedman et al., 2010; Hosmer et al., 2013). The model used a full preprocessing-and-model pipeline within inner five-fold cross-validation, with 20 inverse-penalty values C logarithmically spaced from 10^−4^ to 10^4^ and selection by mean inner-fold ROC AUC. Median imputation and standardization were fitted separately within each inner training partition and refitted on the full outer training partition after tuning. The outer test partition was not used for selection or preprocessing. Random forest used 600 trees and a minimum of five samples per leaf (Breiman, 2001). Histogram-based gradient boosting used maximum depth 3, learning rate 0.05, 400 iterations, and L2 regularization 1.0 (Friedman, 2001). These fixed tree settings were not tuned on the test data. Comparators were the raw bacterial count, the fixed analyzer UTI flag, and logistic regression using nitrite and leukocyte esterase. Complete preprocessing, hyperparameters, random seeds, and the fitted LASSO coefficient, centering and scaling parameters are supplied with the analysis code and **Supplementary Table 3**. All reported model results use this pipeline.

All models and comparators were evaluated using the same outer cross-validation splits and outcome definitions. Bacterial count and the analyzer UTI flag were evaluated directly as predictors, without additional model fitting, and their discrimination was assessed using ROC AUC. Decision curve analysis used the predicted probabilities from the LASSO model. Bacterial-count thresholds were evaluated using the recorded counts in particles/μL.

#### 2.4.2 Sample-size considerations

The sample size was determined by the number of eligible specimens available during the study period. No formal sample-size calculation was performed before data collection. A *post hoc* assessment using the method of Riley et al. (2019), implemented in pmsampsize version 1.1.3, estimated minimum sample sizes of 384 specimens, including 188 culture-positive specimens, for the culture-positivity model and 1,102 specimens, including 243 likely contaminated specimens, for the contamination model. These calculations assumed 18 predictor parameters, a C-statistic of 0.896, and an outcome prevalence of 0.487 for culture positivity, and 11 predictor parameters, a C-statistic of 0.700, and an outcome prevalence of 0.2197 for contamination.

The culture-positivity analysis included 485 specimens from 377 patients, with 236 positive cultures. The contamination analysis included 621 specimens from 477 patients, of which 136 were classified as likely contaminated. The sample-size estimates assumed independent observations and partly relied on observed model performance; they therefore served as approximate benchmarks rather than confirmation of sample-size adequacy. Repeated specimens from individual patients were accounted for in the validation procedure through patient-level grouping. Given the smaller sample and event counts relative to the estimated requirements, contamination modeling was considered exploratory.

#### 2.4.3 Cross-validation and temporal evaluation

Model performance was evaluated using five repeats of stratified, patient-grouped ten-fold cross-validation (random seeds 42–46). The outer ten-fold cross-validation assessed predictive performance, while an inner five-fold cross-validation within each outer training set was used to select the LASSO penalty parameter. At both levels, all specimens from the same patient were assigned to the same fold. Imputation and standardization were fitted within each inner training fold and then refitted on the full outer training set after parameter selection. The six models and comparators were evaluated using identical outer folds. Within each repeat, out-of-fold predictions were pooled across all specimens to calculate a single ROC AUC, with equal weight assigned to each specimen. The primary discrimination measure was the mean of these five AUCs. Predictions from the first repeat (seed 42) were used for ROC curves, calibration assessment, decision curve analysis, and threshold evaluation. A sensitivity analysis included only the earliest eligible specimen from each patient.

An exploratory temporal validation used 1 March 2024 as the cutoff. Models were trained on 351 specimens from 276 patients collected before this date, with patient-grouped five-fold cross-validation used to select the LASSO penalty parameter. Among the 134 later specimens, 20 specimens from 16 patients represented in the training set were excluded, leaving 114 specimens from 101 previously unseen patients for evaluation. All six methods were evaluated on this patient-independent test set. A sensitivity analysis additionally included the 20 overlapping specimens, yielding a total of 134 specimens. Because the cutoff was not documented as prespecified, the temporal validation was considered exploratory.

Confidence intervals were estimated using 2,000 patient-level bootstrap samples. Each sampled patient contributed all their specimens, including repeated contributions when sampled more than once. The same bootstrap samples were used for paired comparisons between methods. For repeated cross-validation, the AUC for each repeat and the mean across repeats were recalculated in every bootstrap sample. Models were not refitted during bootstrapping, so these intervals describe uncertainty conditional on the fitted predictions rather than the full uncertainty of model development. No external validation cohort was available.

#### 2.4.4 Performance measures and retrospective decision analysis

Performance assessment included discrimination, calibration, and decision analysis (Steyerberg et al., 2010; Van Calster et al., 2019). Calibration-in-the-large was estimated with the prediction logit as an offset, and calibration intercept and slope were estimated jointly by logistic recalibration; Brier score measured mean squared probability error. Decision-curve analysis used the action “perform culture” and report-defined positivity as the endpoint, comparing model-guided culture with culture-all and culture-none within the retrospectively defined primary cohort. Following Vickers and Elkin (2006), net benefit at threshold p_t_ was calculated by Equation 1:

(1) $$\text{NB}({\text{p}}_{\text{t}})=\text{TP}/\text{N}-(\text{FP}/\text{N})\times {\text{p}}_{\text{t}}/(1-{\text{p}}_{\text{t}})$$

Here, TP is the number of culture-positive specimens assigned to culture, FP is the number of culture-negative specimens assigned to culture, and N is the number of evaluable primary-cohort specimens. A threshold of 0.10 assigns a weight of 1/9 to performing culture on a culture-negative specimen relative to identifying a culture-positive specimen. This is a hypothetical trade-off, not a measured clinical preference or monetary cost. Net benefit above zero indicates improvement over culture-none; comparison with culture-all is also required. The illustrative probability thresholds were not prospectively selected clinical cut-offs. Deferral proportions, false negatives, and predictive values describe retrospective classification consequences, not observed canceled cultures, missed symptomatic infections, or demonstrated safety. Contamination was known only after culture, so these estimates cannot be applied directly to the full laboratory workflow.

Prevalence scenarios varied the assumed proportion of report-defined culture-positive specimens while holding sensitivity (Se) and specificity (Sp) fixed at the empirically selected high-sensitivity operating point. For an assumed prevalence π, Bayes’ theorem gives

(2) PPV=Seπ/[Seπ+(1−Sp)(1−π)]

and

(3) NPV=Sp(1−π)/[(1−Se)π+Sp(1−π)]

Equations 2 and 3 were used only for projections over π = 0.02–0.50. They assume transportability of sensitivity and specificity, which was not tested; changes in case mix, specimen quality, or analytical conditions may invalidate this assumption. These are neither external-validation results nor estimates of symptomatic-UTI risk.

Continuous variables were summarized as medians and interquartile ranges, and categorical variables as counts and proportions. The cultured versus non-cultured comparison was descriptive at the specimen level. **Table 1** reports distributions and absolute standardized mean differences, without P values, because specimens include repeated patients and the group sizes are highly unequal. For auditability, **Supplementary Table 14** retains the conventional independent-specimen Mann–Whitney and Pearson calculations, explicitly without valid patient-adjusted inference. They are not used to support conclusions about group differences. Mann–Whitney calculations use average ties, tie-adjusted variance and a 0.5 continuity correction; Pearson tests use no continuity correction.

**Table 1:** Characteristics of cultured versus non-cultured urine specimens.

Characteristic	Not cultured	Cultured	SMD
No. of specimens	37,664	643	
Quantitative parameters, median (IQR)			
Bacterial count, particles/µL	12.0 (4.6–61.8)	215.7 (16.2–13336.2)	0.94
White-blood-cell count,/µL	3.3 (1.3–11.4)	53.8 (5.7–345.7)	1.08
Red-blood-cell count,/µL	3.0 (1.2–8.5)	8.5 (2.5–66.3)	0.69
Epithelial-cell count,/µL	2.2 (0.9–5.8)	4.4 (1.9–10.7)	0.46
Squamous epithelial cells,/µL	0.7 (0.1–2.4)	1.2 (0.4–3.9)	0.27
Transitional epithelial cells,/µL	0.0 (0.0–0.1)	0.1 (0.0–0.7)	0.50
Conductivity, mS/cm	14.3 (10.7–18.1)	12.5 (8.9–15.9)	0.36
pH	6.0 (5.5–6.5)	6.0 (5.5–6.5)	0.11
Specific gravity	1.015 (1.011–1.021)	1.013 (1.009–1.018)	0.26
Dipstick positivity and recorded analyzer outputs, n (%)			
Nitrite	1064 (2.8)	172 (26.7)	0.72
Leukocyte esterase	5064 (13.4)	325 (50.5)	0.87
Blood	3665 (9.7)	288 (44.8)	0.86
Protein	2501 (6.6)	199 (30.9)	0.65
Turbidity (cloudy)	1068 (2.8)	141 (21.9)	0.61
Recorded analyzer UTI flag	1474 (3.9)	217 (33.7)	0.83
WBC clumps	1549 (5.0)	224 (36.1)	0.83
Recorded presumptive Gram-negative flag	1262 (3.4)	185 (28.8)	0.74
Recorded presumptive Gram-positive flag	2860 (7.6)	135 (21.0)	0.39
Demographics			
Age, years, median (IQR)	55 (44–65)	60 (51–69)	0.40
Male sex, n (%)	15,624 (46.7)	244 (37.9)	0.18
Female sex, n (%)	17,798 (53.3)	399 (62.1)	

Data are median (interquartile range) or n (%). Absolute standardized mean differences (SMDs) are defined in Equations 4 and 5; no group-comparison P values are presented because repeated specimens violate independent-observation assumptions. Cultured specimens have paired culture (n = 643), including 621 with an available UF bacterial count; non-cultured specimens lack paired culture (n = 37,664). **Supplementary Table S9** gives variable-specific availability and denominators. Continuous summaries, nitrite, WBC clumps and sex use available observations; leukocyte esterase, blood, protein, turbidity and recorded flags use the full group denominator under the archived coding rules. Flag percentages describe recorded target text, not positivity among verified measured results. **Supplementary Table 14** preserves conventional test calculations for audit only, without patient-adjusted inferential validity. IQR, interquartile range.

Absolute standardized mean differences (SMDs) summarized measured selection between 643 cultured and 37,664 non-cultured specimens. For continuous variables, Equation 4 compares group means and pooled standard deviations, with particle counts log(1 + x)-transformed (Austin, 2009):

(4) $$\left|\text{SMD}|\;=\;|{\bar{\text{x}}}_{1}-{\bar{\text{x}}}_{2}|\;/\;\surd [({\text{SD}}_{1}^{2}\;+{\text{SD}}_{2}^{2})/2\right]$$

For binary variables, group proportions p_1_ and p_2_ were compared using Equation 5 (Austin, 2009):

(5) $$\left|\text{SMD}|=|{\text{p}}_{1}-{\text{p}}_{2}|\;/\;\surd \{[{\text{p}}_{1}(1-{\text{p}}_{1})+{\text{p}}_{2}(1-{\text{p}}_{2})]/2\right\}$$

An absolute SMD of 0.1 was used as a descriptive reference, not a test of clinical significance. These comparisons quantify observed differences associated with culture ordering; they do not identify the unobserved culture outcomes or correct verification bias in the non-cultured group.

Model analyses used Python 3.12.3, scikit-learn 1.9.0, NumPy 2.5.1, pandas 3.0.5, and SciPy 1.18.0. R 4.5.1 was used for figures, and the archived sample-size assessment used pmsampsize. A reproducible code package records software versions, seeds, outer-fold assignments, derived results and model parameters. Training and prediction times were recorded in the study computing environment (**Supplementary Table 7**). Training times include inner tuning and refitting for LASSO; prediction times are batch times divided by the number of specimens and include preprocessing and pipeline overhead.

Reporting was guided by TRIPOD and TRIPOD+AI (Collins et al., 2015; Moons et al., 2015; Collins et al., 2024), with a revised item-to-section checklist provided separately. Limitations relevant to PROBAST—including outcome misclassification, patient dependence, selection, and lack of external validation—are discussed explicitly (Wolff et al., 2019).

## 3 Results

### 3.1 Study population and selection of cultured specimens

The 621 specimens with paired culture and available UF bacterial counts came from 477 patients; 98 patients contributed 2 to 7 specimens. Median age was 60 years, and 385 specimens (62.0%) were from female patients. Outcomes were report-defined negative in 249 specimens (40.1%), positive in 236 (38.0%), and likely contaminated in 136 (21.9%). The primary positive-versus-negative cohort comprised 485 specimens from 377 patients (**Figure 1**).

**Figure 1:**
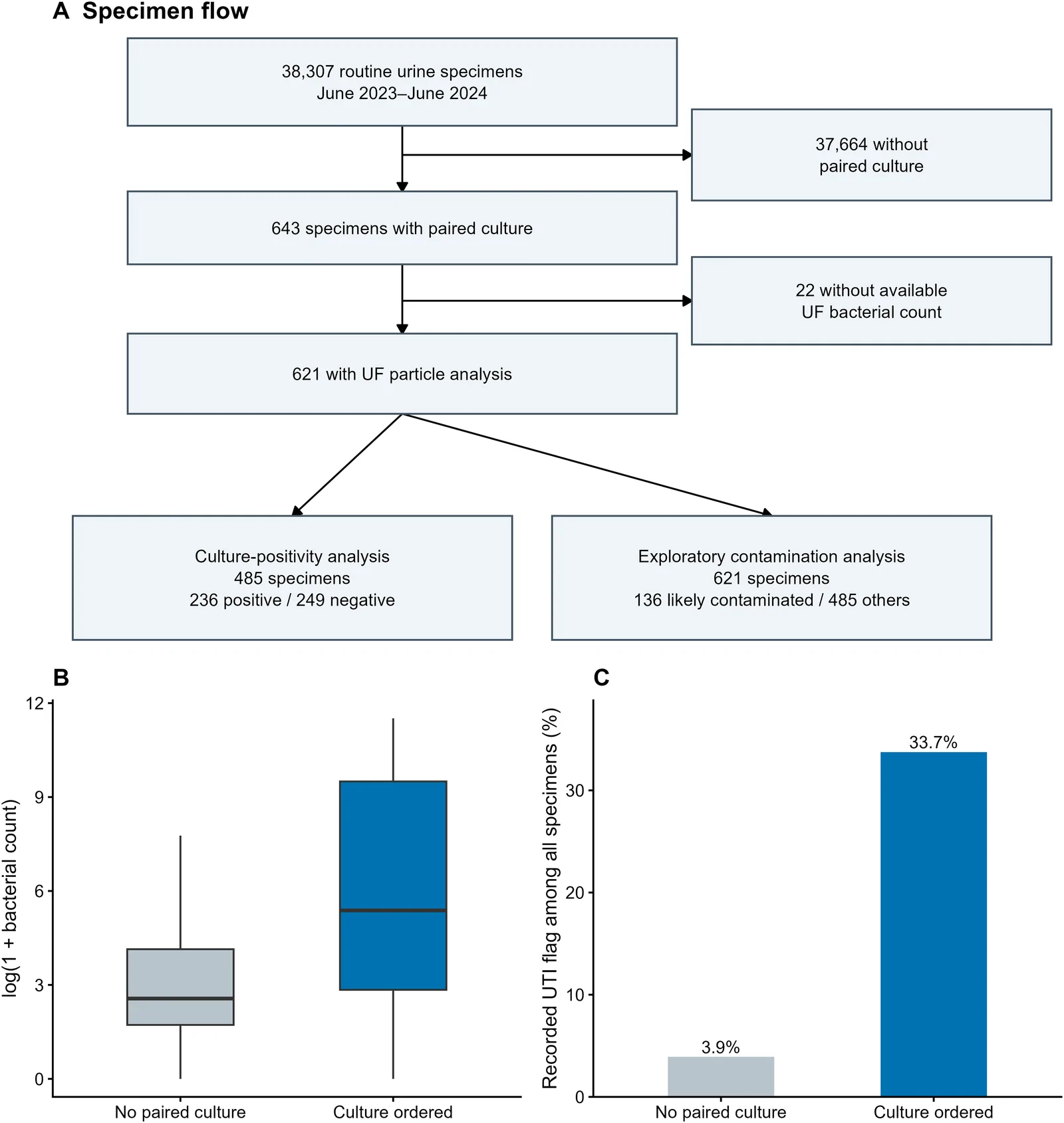
Study specimens and culture-ordering selection. **(A)** Flow from 38,307 unique clinical urinalysis specimens to 643 paired cultures, 621 specimens from 477 patients with an available UF bacterial count, and 485 report-defined positive/negative specimens from 377 patients. The 22 excluded specimens lacked an available UF bacterial count. **(B)** Bacterial count among 31,223 non-cultured and 621 cultured specimens with available values, shown as ln(1 + BACT), with BACT in particles/μL. Boxes span the 25th–75th percentiles, the central line denotes the median, and whiskers extend to the most extreme observations within 1.5 interquartile ranges from the box on the transformed scale; outlier points are not displayed. **(C)** the proportion with recorded UTI-flag text in cultured and non-cultured specimens. These comparisons describe measured selection; the non-cultured group has no reference culture outcome. BACT, bacterial particle count; UF, urine particle analysis; UTI flag, analyzer output rather than a clinical diagnosis.

The 643 cultured specimens differed from the 37,664 non-cultured specimens (**Table 1**; **Figure 1**). Median bacterial count was 215.7 versus 12.0 particles/μL, median white blood cell count was 53.8 versus 3.3 cells/μL, and the proportion with recorded UTI-flag text was 33.7% versus 3.9%. Absolute SMDs were 0.94 for bacterial count, 1.08 for white blood cell count, 0.83 for the UTI flag, and 0.72 for nitrite; age and sex differences were smaller. Presumptive Gram-negative flags occurred in 28.8% versus 3.4% (SMD 0.74), and Gram-positive flags in 21.0% versus 7.6% (SMD 0.39). Among the 485 primary-cohort specimens, report-defined culture positivity was 236/485 (48.7%). Culture outcomes were unavailable in the non-cultured reference group. The larger Gram-negative SMD indicates a greater difference in recorded flag proportions between cultured and non-cultured specimens, supporting selection-associated imbalance. Without culture results in the non-cultured group, these flags cannot establish its true organism distribution or serve as culture-confirmed Gram classifications.

### 3.2 Organism spectrum in report-defined positive cultures

Among the 236 culture-positive specimens, 255 isolates were recovered. Of these, 194 (76.1%) were Gram-negative bacteria, 55 (21.6%) were Gram-positive bacteria, and 6 (2.4%) were yeasts (**Figure 2A**; **Supplementary Table 1**). *Escherichia coli* was the most frequently isolated organism (n = 143), followed by *Enterococcus faecalis* (n = 20), *Klebsiella pneumoniae* (n = 17), *Streptococcus agalactiae* (n = 8), the *Acinetobacter baumannii* report group (n = 7: 3 species-level and 4 complex-level reports), and *Staphylococcus haemolyticus* (n = 7).

**Figure 2:**
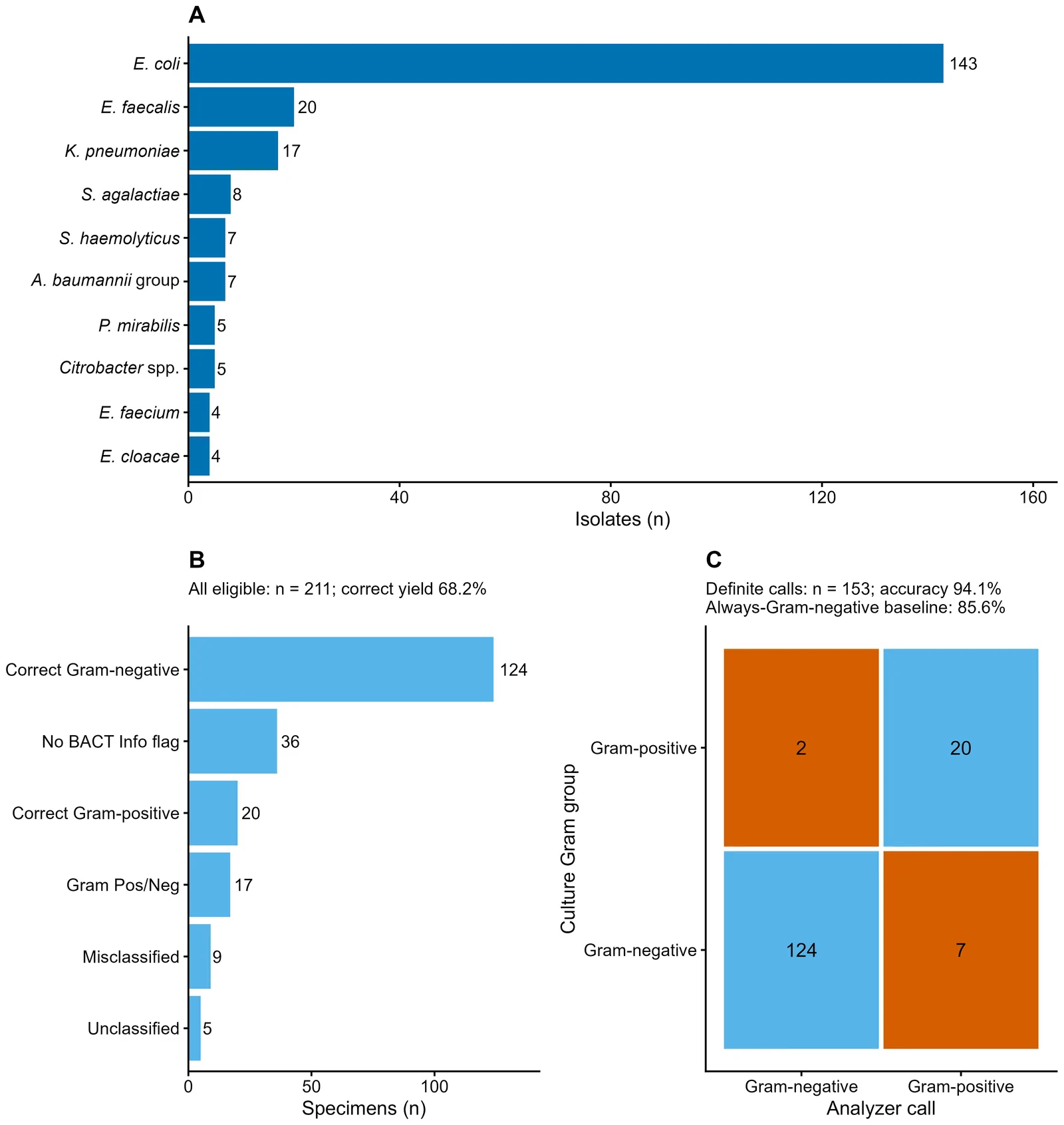
Culture organisms and presumptive Gram classification. **(A)** Leading organisms among 255 isolates from 236 positive specimens. **(B)** Gram-result categories in 211 eligible single-organism Gram-negative/Gram-positive specimens; definite results were available in 153 (72.5%), and correct definite results in 144 (68.2%). **(C)** Confusion matrix restricted to 153 definite calls (rows, culture category; columns, analyzer flag). Accuracy of 94.1% is conditional on a definite result, compared with 85.6% for always predicting Gram-negative on the same subset. Gram Pos/Neg denotes dual Gram-positive and Gram-negative flags (17 specimens). No BACT Info flag denotes no BACT Info alert (36 specimens); Unclassified denotes an indeterminate scattergram classification (5 specimens). These are explicit report categories, not missing exported values.

### 3.3 Prediction of culture positivity and retrospective decision analysis

Mean pooled AUC across five repeats was 0.895 (95% CI 0.866–0.921) for LASSO, 0.897 (95% CI 0.869–0.924) for random forest, and 0.893 (95% CI 0.865–0.919) for gradient boosting; bacterial count alone achieved 0.892 (95% CI 0.864–0.919). Nitrite plus leukocyte esterase and the analyzer UTI flag achieved AUCs of 0.838 and 0.799, respectively. For the paired repeated-CV comparison with bacterial count, ΔAUC was +0.0027 (95% CI -0.0077 to +0.0131) for LASSO, + 0.0053 (95% CI -0.0081 to +0.0192) for random forest, and +0.0012 (95% CI -0.0157 to +0.0185) for gradient boosting. None of these intervals demonstrated superior discrimination over the fixed bacterial-count score. **Supplementary Table 4** separates repeated summaries from individual validation analyses. **Figures 3A, C, D** show the first-repeat ROC, AUC and calibration results; **Figure 4A** shows the corresponding decision analysis.

**Figure 3:**
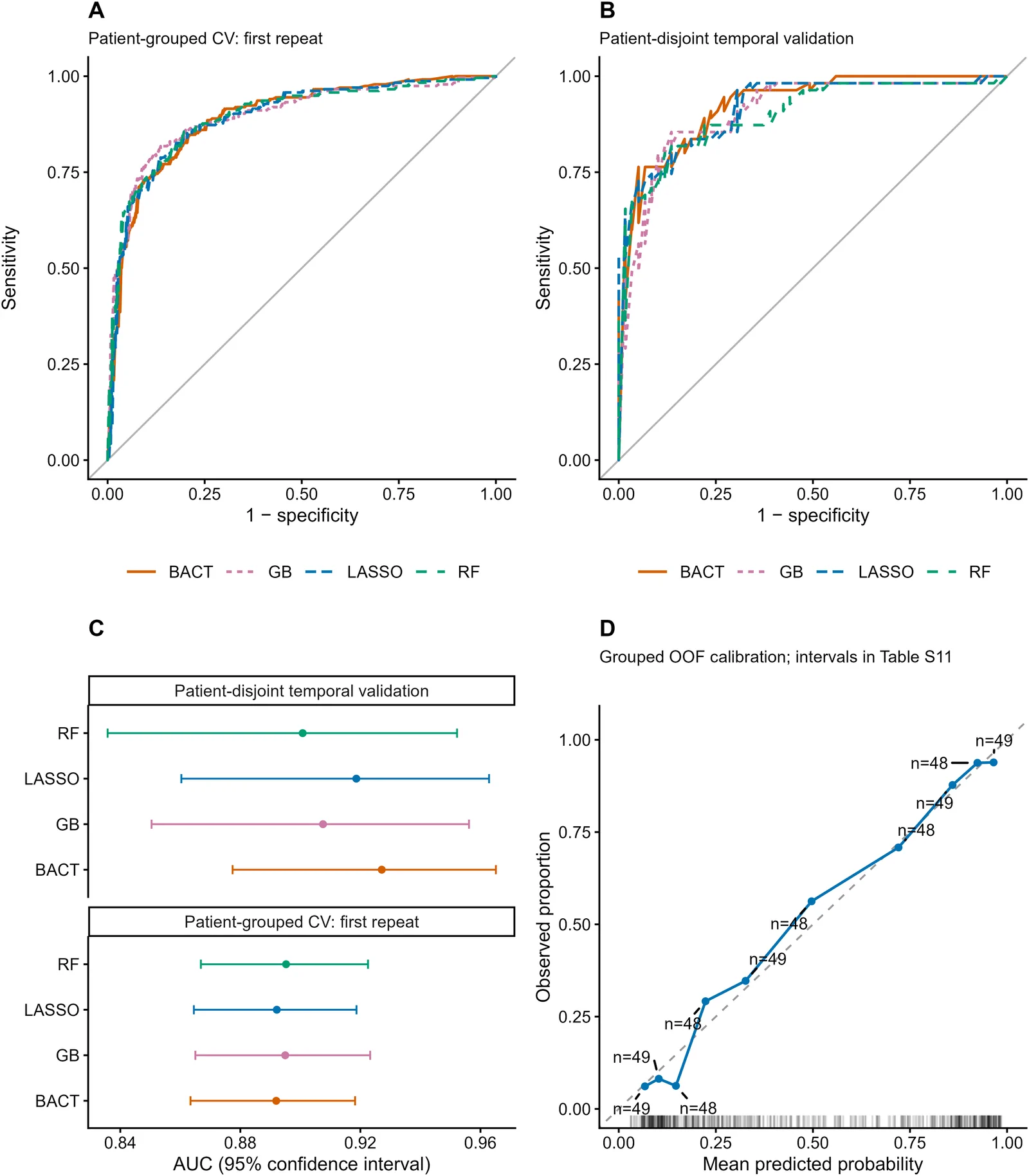
Patient-isolated internal validation of culture-positivity prediction. **(A)** ROC curves from the first patient-grouped ten-fold repeat and **(B)** the 114-specimen patient-disjoint temporal test. **(C)** AUCs and patient-cluster 95% intervals for these two analyses, conditional on stored predictions. **(D)** First-repeat LASSO calibration in prediction quantile bins, labeled with bin sample sizes; the rug shows the prediction distribution and the diagonal ideal calibration. **Supplementary Table S11** reports calibration metric intervals. First-repeat AUCs differ from the mean of five repeat-level AUCs reported in the abstract. Bacterial count is a fixed score; all displayed comparators use identical evaluation specimens. ROC, receiver-operating-characteristic; AUC, area under the ROC curve; OOF, out-of-fold; BACT, bacterial particle count; LASSO, least absolute shrinkage and selection operator; RF, random forest; GB, gradient boosting.

**Figure 4:**
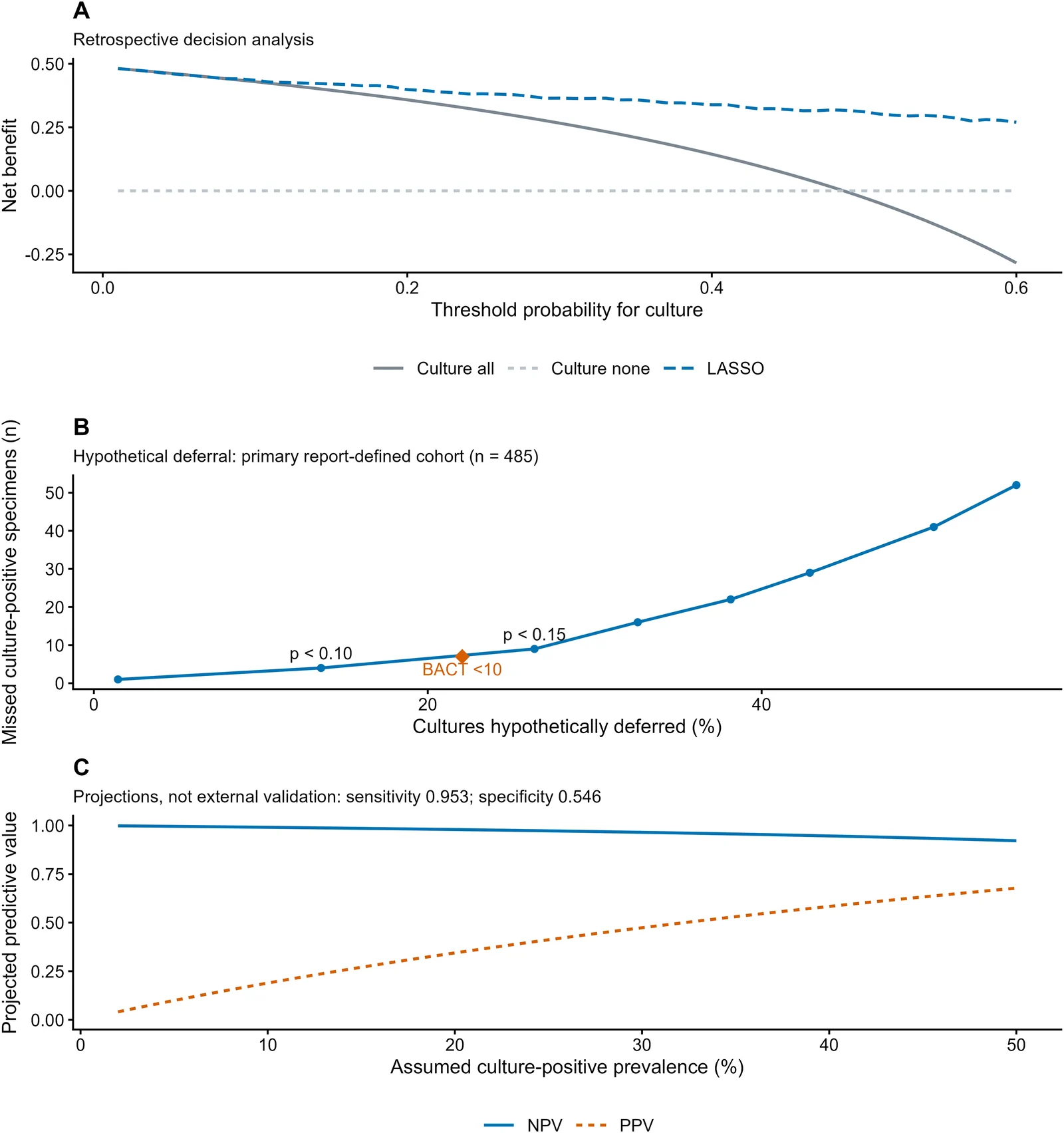
Retrospective decision analyses conditional on the 485-specimen primary report-defined cohort. **(A)** First-repeat LASSO decision curve with culture-all and culture-none comparators. The threshold specifies a hypothetical trade-off for performing culture; net benefit is not a measured treatment effect. **(B)** Hypothetical deferral and missed culture-positive specimens across first-repeat thresholds; BACT <10 particles/μL is a fixed comparator. The contamination outcome used to define this cohort is not known before culture. **(C)** Projected NPV and PPV under varying assumed positivity prevalence with sensitivity 225/236 and specificity 136/249 held fixed at retrospectively selected threshold 0.190064. These are algebraic projections, not observations or external validation. NPV, negative predictive value; PPV, positive predictive value; BACT, bacterial particle count; LASSO, least absolute shrinkage and selection operator.

In the earliest-specimen sensitivity analysis (377 patients; 186 positive reports), LASSO AUC was 0.879 (95% CI 0.841–0.914) and bacterial-count AUC was 0.896 (95% CI 0.863–0.927). Temporal training included 351 specimens from 276 patients, with 171 positive reports. The principal patient-disjoint temporal test contained 114 specimens from 101 patients, with 55 positive reports, after excluding 20 later specimens from 16 patients represented in training. LASSO AUC was 0.919 (95% CI 0.860–0.963), compared with 0.927 (95% CI 0.877–0.965) for bacterial count, 0.901 (95% CI 0.836–0.952) for random forest, and 0.908 (95% CI 0.850–0.956) for gradient boosting. Nitrite/LE and UTI-flag comparisons use the same test subset (**Supplementary Table 4**). Temporal LASSO calibration-in-the-large was 0.006 (95% CI -0.523–0.514), slope 1.081 (95% CI 0.825–1.537), and Brier score 0.120 (95% CI 0.082–0.163) (**Supplementary Table 11**). **Figures 3B, C** displays the patient-disjoint temporal evaluation. The full overlapping 134-specimen time split is only a sensitivity analysis (**Supplementary Table 4**). Training and test predictor distributions and encoded-data availability are described in **Supplementary Table 15**.

The full-cohort LASSO fit selected C = 0.615848 and retained 14 nonzero predictor coefficients. Bacterial count had the largest absolute standardized coefficient. The full-cohort fit was used only to report an executable model specification, not to estimate validation performance (**Supplementary Table 3**). In the first patient-grouped repeat, calibration-in-the-large was 0.026 (95% CI -0.214–0.262); the jointly estimated intercept and slope were 0.033 (95% CI -0.212–0.289) and 1.032 (95% CI 0.878–1.229); Brier score was 0.129 (95% CI 0.111–0.147). Decile counts and probability distributions accompany the calibration plot (**Supplementary Table 12**); **Supplementary Table 11** reports the corresponding metrics for all three fitted multivariable models. Gradient boosting had a first-repeat calibration slope of 0.684 (95% CI 0.577–0.837), consistent with overly extreme predictions, whereas LASSO and random-forest slope intervals included 1. Its temporal slope was 0.702 (95% CI 0.504–1.082), with greater uncertainty. Discrimination close to bacterial count therefore does not imply equally reliable probabilities across models. Decision curves compare hypothetical culture decisions with culture-all and culture-none within the retrospectively report-defined primary cohort (**Figure 4A**).

At first-repeat LASSO probabilities <0.10 and <0.15, 66/485 (13.6%) and 128/485 (26.4%) specimens would have been deferred, including 4 and 9 positive reports; NPVs were 0.939 (0.878–0.987) and 0.930 (0.883–0.969) (patient-cluster 95% CIs). Bacterial count <10 particles/μL would have deferred 107/485 (22.1%), including seven positive reports (NPV 0.935 (0.882–0.976)). In the 114-specimen patient-disjoint temporal test, the same LASSO thresholds would have deferred 15 and 33 specimens, including 1 and 1 positive reports; NPVs were 0.933 (0.812–1.000) and 0.970 (0.909–1.000). **Figure 4B** and **Supplementary Table 2** give the corresponding trade-offs; no cultures were actually withheld.

### 3.4 Prevalence-based projections

Using the first-repeat LASSO predictions, the highest observed threshold that maintained sensitivity ≥0.95 was retrospectively identified as 0.190064. At this threshold, sensitivity was 225/236 (0.953) and specificity was 136/249 (0.546). Deferring culture for specimens with predicted probabilities below this threshold would have deferred 147 specimens, including 11 culture-positive specimens, yielding an NPV of 136/147 (0.925) at the observed culture-positive proportion. With sensitivity and specificity held constant, projected NPVs at assumed culture-positive proportions of 20%, 10%, and 5% were 0.979, 0.991, and 0.996, respectively (**Figure 4C**). These estimates were illustrative projections based on a retrospectively selected threshold, rather than observed results from populations with lower culture-positive proportions.

### 3.5 Presumptive Gram classification

A total of 211 single-organism culture-positive specimens from 173 patients were eligible for Gram classification analysis, including 175 Gram-negative and 36 Gram-positive specimens. A single Gram flag was recorded in 153/211 specimens (72.5%; patient-cluster 95% CI, 66.3–78.5%), both flags in 17/211 (8.1%), no BACT Info flag in 36/211 (17.1%), and an unclassified result in 5/211 (2.4%). The 153 specimens with definite classifications came from 131 patients and comprised 131 culture-confirmed Gram-negative specimens from 112 patients and 22 Gram-positive specimens from 20 patients. Among specimens with definite classifications, the analyzer correctly classified 124 of 131 Gram-negative specimens and 20 of 22 Gram-positive specimens. Seven Gram-negative specimens were classified as Gram-positive, and two Gram-positive specimens were classified as Gram-negative. Accuracy was 144/153 (94.1%; patient-cluster 95% CI, 90.2–97.4%), compared with 131/153 (85.6%) for an always-Gram-negative prediction, an absolute difference of 8.5 percentage points (paired patient-cluster 95% CI, 2.0–15.3 percentage points). Sensitivity and specificity for Gram-negative identification were 124/131 (94.7%) and 20/22 (90.9%), respectively. Balanced accuracy was 92.8%, compared with 50.0% for the always-Gram-negative comparator, and Cohen’s κ was 0.782. Across all 211 eligible specimens, 144 received a correct, definite Gram classification (68.2%; patient-cluster 95% CI, 61.9–74.5%; **Figure 2** and **Supplementary Table 8**). Definite-result coverage was 131/175 (74.9%) among culture-confirmed Gram-negative specimens and 22/36 (61.1%) among Gram-positive specimens. The proportions with a correct, definite classification among all specimens in each category were 124/175 (70.9%) and 20/36 (55.6%), respectively. **Supplementary Table 8** provides patient-cluster confidence intervals for coverage, definite-result accuracy, the paired accuracy difference from the always-Gram-negative comparator and correct definite-result yield, together with counts of dual-flag, unclassified and no-BACT-Info-flag results. Its Wilson intervals for conditional sensitivity and specificity are descriptive and do not adjust for repeated specimens from the same patient.

### 3.6 Sensitivity analyses

The 236 positive specimens comprised 211 single-bacterial-organism specimens, 19 two-organism specimens, and 6 yeast-only specimens, yielding 255 isolates. Four *post hoc* sensitivity analyses used patient-grouped validation. Excluding yeast-only cultures gave 479 specimens (230 positives), with LASSO AUC 0.899 and bacterial-count AUC 0.895; excluding two-organism cultures gave 466 specimens (217 positives), with corresponding AUCs 0.897 and 0.894. Reclassifying the 20 species-rule likely-contamination reports as negative yielded 505 specimens (236 positives), with AUCs 0.883 and 0.883. Assigning the same 20 reports to the positive category instead gave 505 specimens (256 positives), with LASSO AUC 0.883 (95% CI 0.853–0.911) and bacterial-count AUC 0.883 (95% CI 0.852–0.910). These deliberately extreme alternative assignments assess label dependence; neither adjudicates infection. The exploratory contamination model achieved grouped AUC 0.701 (95% CI 0.647–0.753), average precision 0.367 against an event prevalence of 0.219, and Brier score 0.159. Squamous epithelial cell count alone had AUC 0.640. At a contamination-probability threshold ≥0.20, sensitivity was 0.846, specificity 0.423, precision 0.291, and F1 score 0.433. Its limited predictive performance does not support discarding specimens or a sequential culture-deferral pathway (**Supplementary Tables 5**, **6**; **Figures 5**–**7**).

**Figure 5:**
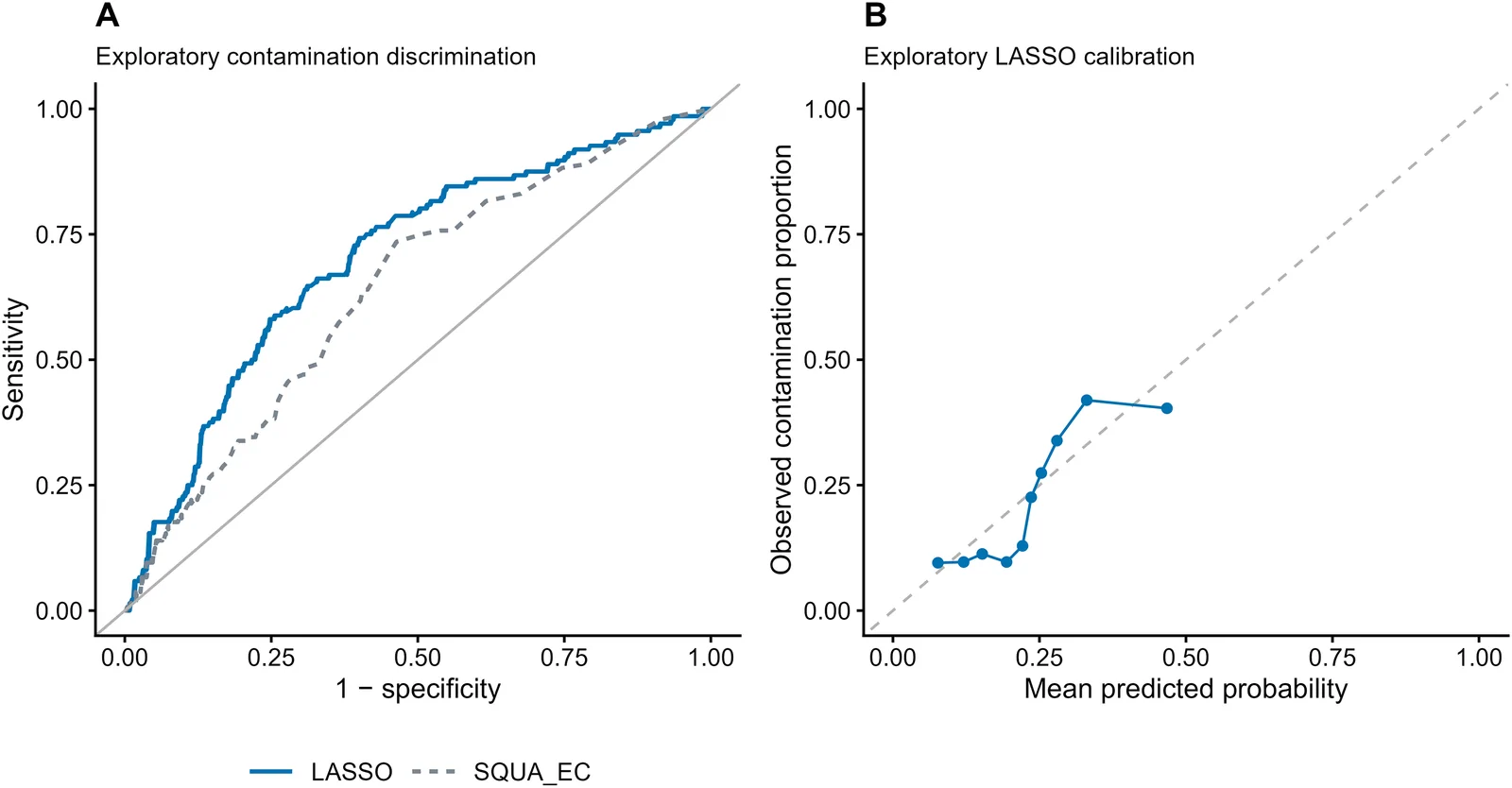
Exploratory contamination prediction. **(A)** Patient-grouped out-of-fold ROC curves for LASSO and squamous epithelial cell count. **(B)** LASSO decile calibration. N = 621, including 136 likely-contamination labels. The diagonal in panel B indicates ideal calibration. ROC, receiver operating characteristic; LASSO, least absolute shrinkage and selection operator; SQUA_EC, squamous epithelial cell count.

**Figure 6:**
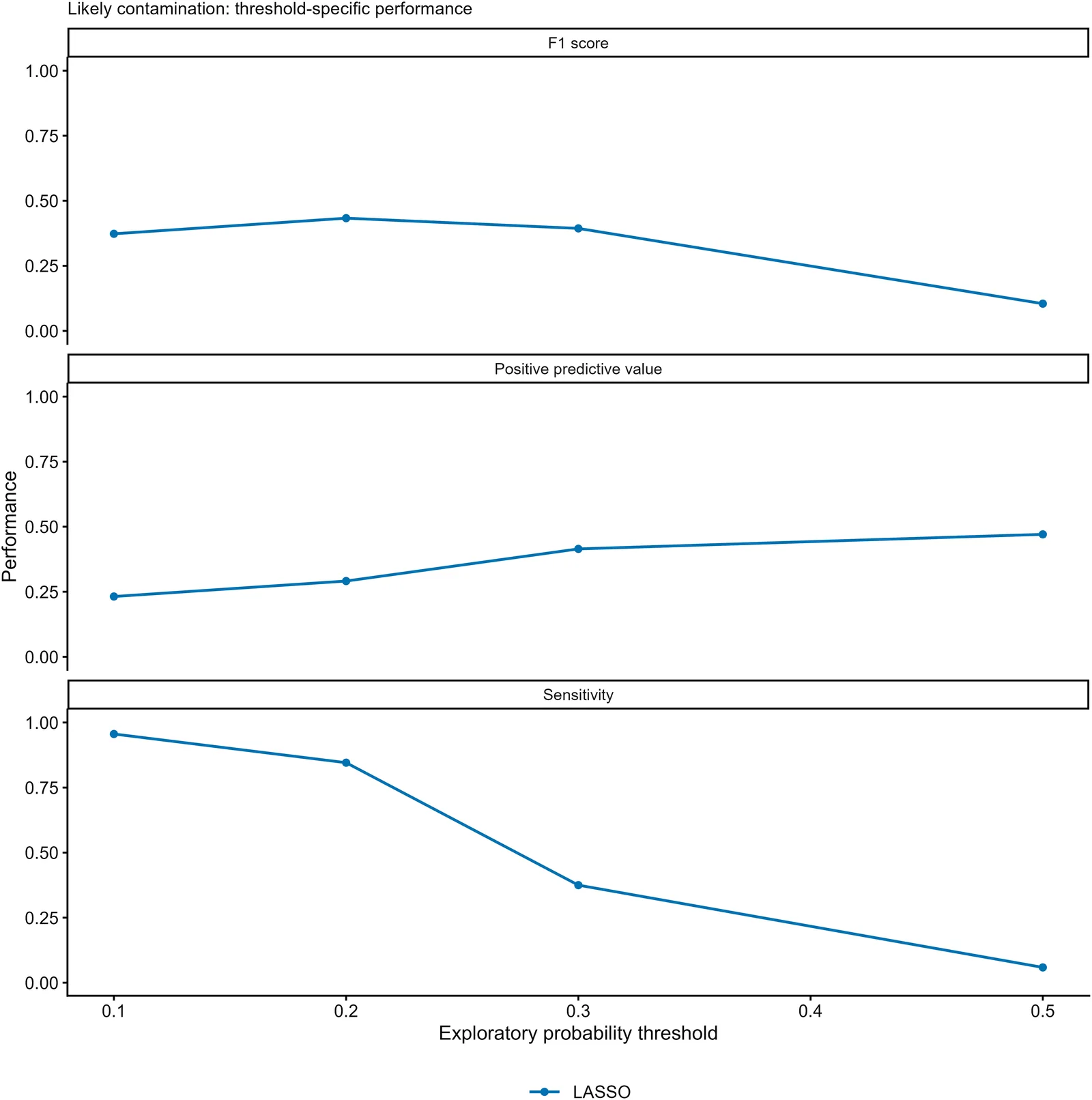
Exploratory contamination threshold trade-offs. Sensitivity, precision (PPV) and F1 at predicted probabilities 0.10,0.20,0.30 and 0.50, from the same patient-grouped predictions. Lines connect evaluated points for visual guidance; no threshold is recommended for use. LASSO, least absolute shrinkage and selection operator; PPV, positive predictive value.

**Figure 7:**
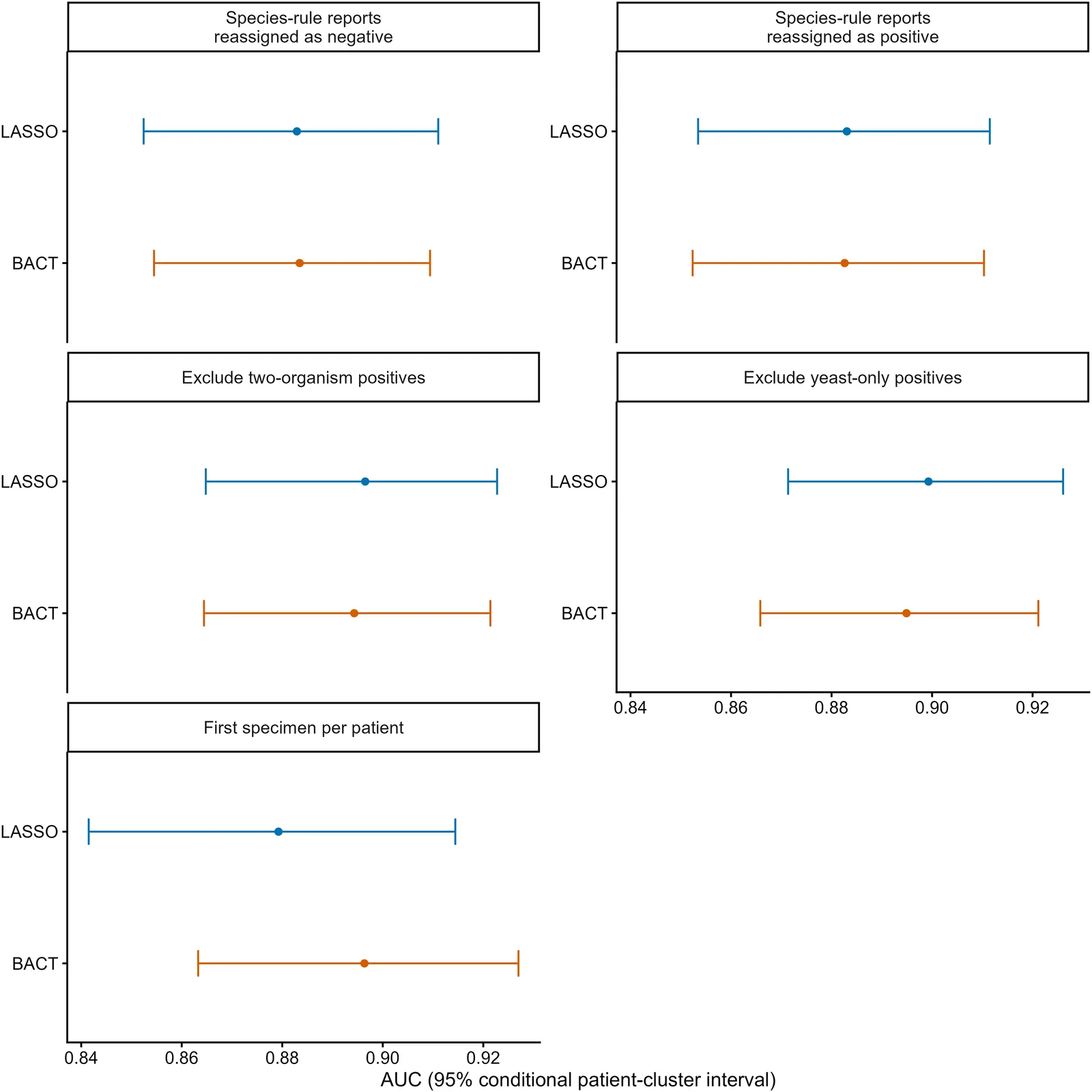
Patient-grouped sensitivity analyses: earliest specimen per patient and four alternative outcome definitions. Points show AUC and 95% patient-cluster intervals conditional on fitted predictions. Organism-text reports are assigned to negative or positive only in alternative scenarios, not as clinical adjudication. BACT, bacterial count; LASSO, least absolute shrinkage and selection operator; AUC, area under the receiver-operating-characteristic curve.

## 4 Discussion

The principal finding of this study was that bacterial count alone provided discrimination of urine culture positivity close to that of the multivariable models. Mean AUCs ranged from 0.892 to 0.897, and paired comparisons showed no clear improvement over bacterial count. One possible explanation is that bacterial count already captures much of the information relevant to culture positivity, leaving limited additional information for other urinalysis variables to contribute. These findings emphasize the importance of comparing complex prediction models with simple, readily available laboratory indicators.

Previous studies have evaluated automated urinalysis and machine learning for predicting urine culture results in different clinical settings. Burton et al. (2019) compared machine-learning models with microscopy-based measures, while Taylor et al. (2018) investigated symptomatic emergency-department patients. Studies of the UF series have also differed in their culture workflows: Korsten et al. (2024) routed oncology and intensive-care specimens directly to culture, whereas Knežević et al. (2026) used leukocyte-guided culture during the main study phase. In an oncology cohort, Laracy et al. (2025) evaluated 58,098 paired urine cultures and dipstick tests, incorporating neutropenia and inpatient antibiotic exposure. Differences in populations, culture selection, and outcome definitions limit direct numerical comparisons across studies. Our study complements this literature by evaluating the incremental discrimination of multivariable models over bacterial count within the same cohort and validation framework.

The findings also illustrate the distinction between discrimination and probability calibration. Although the models had similar AUCs, gradient boosting produced more extreme predicted probabilities, as indicated by its first-repeat calibration slope below 1. This matters when predicted probabilities are used to guide decisions at specific thresholds. The retrospective threshold analyses described the balance between the proportion of cultures potentially deferred and the number of culture-positive specimens missed. The prevalence projections further illustrated that NPV increases as culture positivity decreases when sensitivity and specificity remain constant. Application in another population would require validation of both model performance and the proposed thresholds.

Analyzer-derived Gram classification provided useful additional information when a definite result was recorded. Accuracy among these specimens was 94.1%, compared with 85.6% for an always-Gram-negative prediction. However, definite results were available for 72.5% of eligible specimens, and 68.2% of all eligible specimens received a correct, definite classification. Coverage was lower for Gram-positive than for Gram-negative specimens, highlighting the importance of reporting both accuracy and result availability. These findings support further evaluation of presumptive Gram information as an adjunct to laboratory assessment, including its availability before definitive culture results and the consequences of incorrect classifications. Estimates for Gram-positive specimens were less precise because only 22 had definite results.

Prediction of likely contamination was more challenging. The exploratory model showed modest discrimination, and its low precision at the evaluated threshold limited its usefulness for identifying specimens that might require recollection. We retained the observed class distribution because over- or undersampling does not necessarily improve discrimination and may adversely affect probability calibration (van den Goorbergh et al., 2022). The limited number of contamination events and the uncertainty of organism-based classifications suggest that future studies should incorporate more detailed specimen-collection information and a stronger reference assessment of contamination.

Strengths of this study include comparison with simple laboratory indicators, patient-grouped nested cross-validation, and temporal evaluation in patients not represented in the training set. Keeping repeated specimens from each patient together reduced the risk of information leakage between training and test data. Evaluation of calibration, threshold performance, and Gram-result coverage provided information beyond AUC alone. The accompanying analysis code, model specification, and aggregate results support reproducibility.

Several limitations should be considered. This was a single-center retrospective study using one analyzer combination, without external validation. Cultured specimens had higher bacterial and white blood cell counts than non-cultured specimens, indicating selection within the laboratory workflow. The primary analysis also excluded likely contaminated specimens on the basis of subsequent culture results. Its 48.7% culture-positive proportion and retrospective decision estimates therefore apply to this selected cohort, rather than the full population of incoming urine specimens. The rationale for the temporal cutoff was not documented, so temporal findings remain exploratory. Bootstrap confidence intervals were calculated from stored predictions and did not include the full uncertainty associated with repeating model development.

The available clinical and laboratory records also limited outcome interpretation. Culture positivity was assessed from laboratory reports, while symptoms, antimicrobial exposure, neutropenia, and clinical outcomes were unavailable. Consequently, symptomatic UTI and asymptomatic bacteriuria could not be distinguished (Nicolle et al., 2019). Organism-based contamination classifications may have misclassified some clinically relevant isolates, and routine culture interpretation may have incorporated urinalysis findings. Sensitivity analyses examined the effect of alternative outcome assignments but could not resolve their clinical significance.

Future prospective studies should enroll the full intended population, document specimen quality and clinical context, and evaluate predefined culture-triage thresholds. Such studies could establish whether bacterial count and presumptive Gram information improve laboratory workflow while maintaining acceptable detection of clinically important culture results. The implementation timings in **Supplementary Table 7** provide a computational benchmark for the study environment. A bacterial-count threshold requires only a numerical comparison, whereas fitted models require preprocessing and prediction. No smart-device deployment was tested; these timings do not establish device-specific latency or predictive performance. Integration would require verification of input compatibility and numerical implementation, followed by validation and assessment of calibration in the intended population.

## 5 Conclusion

Automated urinary bacterial particle count provided good discrimination of urine culture positivity in this cancer-hospital cohort, with no demonstrated improvement from multivariable models. Analyzer-derived presumptive Gram classification achieved high accuracy among specimens with a definite result, although definite results were available for only a subset of eligible specimens. These findings highlight bacterial count as a useful benchmark for evaluating more complex prediction models and support further prospective evaluation of automated urinalysis for urine-culture triage.

## Data Availability

The de-identified analysis dataset is available from the corresponding author on reasonable request, subject to applicable institutional data-access requirements.

## References

[B1] Austin P. C. (2009). Balance diagnostics for comparing the distribution of baseline covariates between treatment groups in propensity-score matched samples. Stat. Med. 28, 3083–3107. doi: 10.1002/sim.3697 19757444 PMC3472075

[B2] Bekeris L. G. Jones B. A. Walsh M. K. Wagar E. A. (2008). Urine culture contamination: a College of American Pathologists Q-Probes study of 127 laboratories. Arch. Pathol. Lab. Med. 132, 913–917. doi: 10.5858/2008-132-913-UCCACO 18517272

[B3] Berger B. Lukey J. Jinadatha C. Navarathna D. H. (2025). Impact of discontinuing automatic reflex urine culture after urinalysis: a diagnostic and antibiotic stewardship initiative. Front. Cell. Infect. Microbiol. 15, 1572936. doi: 10.3389/fcimb.2025.1572936 41141594 PMC12549692

[B4] Breiman L. (2001). Random forests. Mach. Learn. 45, 5–32. doi: 10.1023/A:101093340432441886696

[B5] Broeren M. A. C. Bahçeci S. Vader H. L. Arents N. L. A. (2011). Screening for urinary tract infection with the Sysmex UF-1000i urine flow cytometer. J. Clin. Microbiol. 49, 1025–1029. doi: 10.1128/JCM.01669-10 21248088 PMC3067737

[B6] Burton R. J. Albur M. Eberl M. Cuff S. M. (2019). Using artificial intelligence to reduce diagnostic workload without compromising detection of urinary tract infections. BMC Med. Inform. Decis. Mak. 19, 171. doi: 10.1186/s12911-019-0878-9 31443706 PMC6708133

[B7] Chang K.-W. Tsai C.-Y. Wong S.-M. Chueh J. S.-C. Chang S.-J. (2026). Diagnostic accuracy of urine flow cytometry (UF) in urinary tract infection (UTI) detection and management: a systematic review and meta-analysis. Diagnostics 16, 1275. doi: 10.3390/diagnostics16091275 42121979 PMC13163317

[B8] Claeys K. C. Zhan M. Pineles L. Lydecker A. Clore G. Goto M. . (2021). Conditional reflex to urine culture: evaluation of a diagnostic stewardship intervention within the Veterans' Affairs and Centers for Disease Control and Prevention Practice-Based Research Network. Infect. Control Hosp. Epidemiol. 42, 176–181. doi: 10.1017/ice.2020.400 32838829 PMC8507330

[B9] Collins G. S. Moons K. G. M. Dhiman P. Riley R. D. Beam A. L. Van Calster B. . (2024). TRIPOD+AI statement: updated guidance for reporting clinical prediction models that use regression or machine learning methods. BMJ 385, e078378. doi: 10.1136/bmj-2023-078378 38626948 PMC11019967

[B10] Collins G. S. Reitsma J. B. Altman D. G. Moons K. G. M. (2015). Transparent Reporting of a multivariable prediction model for Individual Prognosis Or Diagnosis (TRIPOD): the TRIPOD statement. BMJ 350, g7594. doi: 10.1136/bmj.g7594 25569120

[B11] De Rosa R. Grosso S. Lorenzi G. Bruschetta G. Camporese A. (2018). Evaluation of the new Sysmex UF-5000 fluorescence flow cytometry analyser for ruling out bacterial urinary tract infection and for prediction of Gram-negative bacteria in urine cultures. Clin. Chim. Acta 484, 171–178. doi: 10.1016/j.cca.2018.05.047 29803898

[B12] Devillé W. L. J. M. Yzermans J. C. van Duijn N. P. Bezemer P. D. van der Windt D. A. W. M. Bouter L. M. (2004). The urine dipstick test useful to rule out infections: a meta-analysis of the accuracy. BMC Urol. 4, 4. doi: 10.1186/1471-2490-4-4 15175113 PMC434513

[B13] Flores-Mireles A. L. Walker J. N. Caparon M. Hultgren S. J. (2015). Urinary tract infections: epidemiology, mechanisms of infection and treatment options. Nat. Rev. Microbiol. 13, 269–284. doi: 10.1038/nrmicro3432 25853778 PMC4457377

[B14] Foxman B. (2014). Urinary tract infection syndromes: occurrence, recurrence, bacteriology, risk factors, and disease burden. Infect. Dis. Clin. North. Am. 28, 1–13. doi: 10.1016/j.idc.2013.09.003 24484571

[B15] Friedman J. H. (2001). Greedy function approximation: a gradient boosting machine. Ann. Stat. 29, 1189–1232. doi: 10.1214/aos/1013203451

[B16] Friedman J. Hastie T. Tibshirani R. (2010). Regularization paths for generalized linear models via coordinate descent. J. Stat. Software 33, 1–22. doi: 10.18637/jss.v033.i01 PMC2929880 20808728

[B17] Hosmer D. W. Lemeshow S. Sturdivant R. X. (2013). Applied Logistic Regression, 3rd Edn (Hoboken, NJ: Wiley). doi: 10.1002/9781118548387

[B18] Jolkkonen S. Paattiniemi E.-L. Kärpänoja P. Sarkkinen H. (2010). Screening of urine samples by flow cytometry reduces the need for culture. J. Clin. Microbiol. 48, 3117–3121. doi: 10.1128/JCM.00617-10 20592157 PMC2937741

[B19] Kim S. Y. Park Y. Kim H. Kim J. Koo S. H. Kwon G. C. (2018). Rapid screening of urinary tract infection and discrimination of Gram-positive and Gram-negative bacteria by automated flow cytometric analysis using Sysmex UF-5000. J. Clin. Microbiol. 56, e02004-17. doi: 10.1128/JCM.02004-17 29769277 PMC6062802

[B20] Knežević D. Travar M. Stojisavljević Đ. Jović D. Grujičić M. (2026). Multivariable urine flow cytometry–based screening for prediction of urine culture positivity. Diagnostics 16, 1022. doi: 10.3390/diagnostics16071022 41975735 PMC13073749

[B21] Korsten K. de Gier A. Leenders A. Wever P. C. Kolwijck E. (2024). Using the Sysmex UF-4000 urine flow cytometer for rapid diagnosis of urinary tract infection in the clinical microbiological laboratory. J. Clin. Lab. Anal. 38, e25004. doi: 10.1002/jcla.25004 38454622 PMC10959182

[B22] Laracy J. C. Chan J. L. Kodama R. Yan J. Raible K. M. Sepkowitz K. . (2025). Improving urinary tract infection diagnostics in oncology: reliability of reflex urine culture in immunosuppressed neutropenic and non-neutropenic cancer patients. Clin. Infect. Dis. 80, 969–974. doi: 10.1093/cid/ciaf018 39812021 PMC12499952

[B23] Liu P. Ban C. Wang J. Zeng Q. Chen M. Wang L. . (2024). Enhancing clinical decision-making: Sysmex UF-5000 as a screening tool for bacterial urinary tract infection in children. PloS One 19, e0304286. doi: 10.1371/journal.pone.0304286 38865329 PMC11168643

[B24] Moons K. G. M. Altman D. G. Reitsma J. B. Ioannidis J. P. A. Macaskill P. Steyerberg E. W. . (2015). Transparent Reporting of a multivariable prediction model for Individual Prognosis Or Diagnosis (TRIPOD): explanation and elaboration. Ann. Intern. Med. 162, W1–W73. doi: 10.7326/M14-0698 25560730

[B25] National Health Commission of the People's Republic of China (2024). Guidelines for the Testing of Urine Specimens in the Clinical Microbiology Laboratory (WS/T 489-2024) (Beijing: National Health Commission of the People's Republic of China).

[B26] Nicolle L. E. Gupta K. Bradley S. F. Colgan R. DeMuri G. P. Drekonja D. . (2019). Clinical practice guideline for the management of asymptomatic bacteriuria: 2019 update by the Infectious Diseases Society of America. Clin. Infect. Dis. 68, e83–e110. doi: 10.1093/cid/ciy1121 30895288

[B27] Penney J. A. Rodday A. M. Sebastiani P. Snydman D. R. Doron S. (2023). Effecting the culture: impact of changing urinalysis with reflex to culture criteria on culture rates and outcomes. Infect. Control Hosp. Epidemiol. 44, 210–215. doi: 10.1017/ice.2022.178 35924370

[B28] Riley R. D. Snell K. I. E. Ensor J. Burke D. L. Harrell F. E. J. Moons K. G. M. . (2019). Minimum sample size for developing a multivariable prediction model: Part II - binary and time-to-event outcomes. Stat. Med. 38, 1276–1296. doi: 10.1002/sim.7992 30357870 PMC6519266

[B29] Steyerberg E. W. Vickers A. J. Cook N. R. Gerds T. Gonen M. Obuchowski N. . (2010). Assessing the performance of prediction models: a framework for traditional and novel measures. Epidemiology 21, 128–138. doi: 10.1097/EDE.0b013e3181c30fb2 20010215 PMC3575184

[B30] Taylor R. A. Moore C. L. Cheung K.-H. Brandt C. (2018). Predicting urinary tract infections in the emergency department with machine learning. PloS One 13, e0194085. doi: 10.1371/journal.pone.0194085 29513742 PMC5841824

[B31] Tibshirani R. (1996). Regression shrinkage and selection via the lasso. J. R. Stat. Soc Ser. B. Stat. Methodol. 58, 267–288. doi: 10.1111/j.2517-6161.1996.tb02080.x40046247

[B32] Van Calster B. McLernon D. J. van Smeden M. Wynants L. Steyerberg E. W. (2019). Calibration: the Achilles heel of predictive analytics. BMC Med. 17, 230. doi: 10.1186/s12916-019-1466-7 31842878 PMC6912996

[B33] van den Goorbergh R. van Smeden M. Timmerman D. Van Calster B. (2022). The harm of class imbalance corrections for risk prediction models: illustration and simulation using logistic regression. J. Am. Med. Inform. Assoc. 29, 1525–1534. doi: 10.1093/jamia/ocac093 35686364 PMC9382395

[B34] Vickers A. J. Elkin E. B. (2006). Decision curve analysis: a novel method for evaluating prediction models. Med. Decis. Making 26, 565–574. doi: 10.1177/0272989X06295361 17099194 PMC2577036

[B35] Wang H. Han F. F. Wen J. X. Yan Z. Han Y. Q. Hu Z. D. . (2023). Accuracy of the Sysmex UF-5000 analyzer for urinary tract infection screening and pathogen classification. PloS One 18, e0281118. doi: 10.1371/journal.pone.0281118 36724192 PMC9891513

[B36] Wolff R. F. Moons K. G. M. Riley R. D. Whiting P. F. Westwood M. Collins G. S. . (2019). PROBAST: a tool to assess the risk of bias and applicability of prediction model studies. Ann. Intern. Med. 170, 51–58. doi: 10.7326/M18-1376 30596875

