# Supplementary material for: Automated urinalysis for predicting urine culture positivity and presumptive Gram classification: a retrospective study at a cancer hospital
Source: Front Cell Infect Microbiol. 2026 Oct 12;16:1935652. doi: 10.3389/fcimb.2026.1935652 (PMC13696273; doi:10.3389/fcimb.2026.1935652)
Supplement: Supplementary file 1 [file SupplementaryFile1.docx]

Supplementary Material

# Supplementary Methods

## Report-based outcome coding

The research endpoint was the classification of archived laboratory reports, not a clinical adjudication of infection. For clean-catch midstream urine yielding a single pathogen, the laboratory’s reporting thresholds during the study period for diagnostically significant growth were ≥10⁵ CFU/mL for Gram-negative bacteria and ≥10⁴ CFU/mL for Gram-positive bacteria. Bacterial cultures classified as positive met the applicable laboratory thresholds, as confirmed by the investigators. The 19 two-organism positive cultures contained two recognized urinary pathogens, each meeting its applicable quantitative threshold. Yeast growth was identified and reported as positive under the laboratory reporting policy. The original analysis labels were retained. Exact precedence in the archived algorithm was: (1) no-growth or <1000 report text → negative; (2) explicitly contaminated or three-or-more-organism mixed-growth text → likely contaminated; (3) single-organism report matching the exact study species rule → likely contaminated; (4) other single-organism or two-organism growth, including yeast → positive.

The negative label does not mean absolute sterility: low-count reports were included by definition. Two-organism and yeast-only positive reports were retained because they met the laboratory definition of a positive culture; this does not establish clinical bacterial co-infection or fungal UTI. Bacterial particle count does not directly quantify yeast growth; separate exclusions of yeast-only and two-organism reports assess this heterogeneity (Table S6).

The organism-text rule was applied to the original Chinese laboratory reports. Table S13 gives English descriptions, Latin names, and the corresponding matching modes. Exact source strings remain in the archived analysis code; the English descriptions are not replacement search strings. Reports containing the Chinese term for Streptococcus were excluded from the non-streptococcal substring match. This rule is narrower than excluding all coagulase-negative staphylococci: single Staphylococcus haemolyticus reports, for example, remained positive. Of 136 likely-contamination labels, 116 came from explicit mixed/contamination text and 20 from the organism-text rule. The generic corynebacterial substring can match reports beyond the individually listed taxa. This is a text-based classification rule, not a clinical assertion that every member of a taxon is contamination. Clinical relevance cannot be adjudicated from this export alone. The investigators confirmed the species-name mappings for T09 and T11, as documented in Table S13.

For organism counting and sensitivity analyses, CFU/mL or CFU/ML unit annotations were removed before splitting on punctuation. This correction retains the one report translated as “Gram-positive bacillus >10^4 CFU/mL” as a single Gram-positive organism rather than two organisms. The main positive label is unchanged. The corrected positive composition is 211 single-bacterial-organism specimens, 19 two-organism specimens and 6 yeast-only specimens; 255 isolates result. The eligible Gram-analysis denominator is 211, including the generic Gram-positive report whose analyzer gave no definite call.

Routine laboratory interpretation could take clinical information and urinary leukocytes into account, according to the author’s clarification of practice. Those consultations and quantitative results were not uniformly available in the research export. Neither independent outcome assessment nor blinded clinical adjudication is established. The computational label mapping itself uses report text, but possible incorporation bias in the underlying reports remains a limitation.

## Predictor dictionary and preprocessing

| **Encoded field** | **Definition** |
| --- | --- |
| BACT_log | Bacterial particle count, particles/μL; log(1+max(x,0)) |
| WBC_log | White blood cell count, cells/μL; log(1+max(x,0)) |
| RBC_log | Red blood cell count, cells/μL; log(1+max(x,0)) |
| EC_log | Epithelial cell count, cells/μL; log(1+max(x,0)) |
| COND_log | Conductivity, mS/cm; log(1+max(x,0)) |
| NIT | Nitrite: negative 0, positive 1 |
| LEU | Leukocyte esterase ordinal: negative 0, trace 0.5, 1+ through 4+ coded 1–4 |
| BLD | Dipstick blood: same ordinal coding |
| PRO | Dipstick protein: same ordinal coding |
| PH | Numeric pH |
| SG | Numeric specific gravity |
| CLOUD | Clear 0, slightly turbid 1, turbid 2 |
| WBC_CLUMPS | Absent 0, present 1; instrument not-measured tokens are missing |
| UTI_flag | Presence of the instrument UTI text flag 1; absence 0 Recorded target-text presence; absent or blank text encoded 0. Measured-negative status is not established for all records. |
| BACT_gram_neg | Gram-negative descriptor present 1; absent 0 No-alert and Unclassified descriptors are coded 0 for both Gram indicators; neither denotes culture negativity or missing data. Other blank source values are not reinterpreted. |
| BACT_gram_pos | Gram-positive descriptor present 1; absent 0 No-alert and Unclassified descriptors are coded 0 for both Gram indicators; neither denotes culture negativity or missing data. Other blank source values are not reinterpreted. |
| age | Age in years |
| sex_male | Male 1, female 0; other entries missing |

Numeric comparator inequalities (<, >) were parsed to their reported numerical boundary by the archived data-preparation code; censored-boundary measurements were not modeled as intervals. Missing values in the fitted predictors were imputed using the training-partition median. Training-partition means and standard deviations were used for LASSO standardization. Preprocessing was inside the inner search during penalty tuning. Fixed bacterial-count and UTI-flag AUCs require no fitting. The nitrite-plus-leukocyte-esterase comparator used imputation, standardization and L2 logistic regression with C=1. A source-token audit found 54 >100000 BACT records among 621 UF-available specimens, including 53/485 primary specimens (49 positive, 4 negative). All 643 paired culture records contained the same 54 such tokens. No other audited numeric field contained an inequality and no negative numeric value occurred (Table S16). Because these BACT observations form the top score block, every possible ordering of its unknown true values gives a bacterial-count AUC between 0.890281 and 0.893617 (recorded-boundary AUC 0.891949). The maximum absolute change is 0.001668: the 49 × 4 censored positive–negative pairs can contribute from 0 to 196 instead of 98 concordance credits, divided by 236 × 249 total pairs. These are deterministic rank bounds, not confidence intervals or imputed concentrations. The BACT <10 classifications are unchanged. Performance of models trained on unavailable uncensored concentrations cannot be inferred from these bounds; reported models use the available boundary-coded inputs and were not refitted for this audit.

The exploratory contamination model used 11 predictors: log-transformed squamous epithelial, total epithelial, transitional epithelial, bacterial and white blood cell counts; the two presumptive Gram flags; turbidity; ordinal color (colorless 0, pale yellow 1, yellow 2, dark yellow 3); age; and male sex. Particle units are cells/μL, except bacteria in particles/μL. Its outer and inner cross-validation grouped by patient. No weighting, oversampling, undersampling or synthetic observations were used.

## Validation analyses

Primary validation assessed new patients in the same culture-ordered setting. All specimens from a patient remained together in every outer and inner partition. Five stratified patient-grouped ten-fold partitions used seeds 42–46; the headline estimand was the mean of five pooled out-of-fold AUCs, with each specimen weighted equally. The same outer folds were used for all six comparators. Each repeat supplied one prediction per specimen; this is not an average of 50 fold-level AUCs. The fixed first repeat (seed 42) supplied ROC curves, calibration, decision curves and threshold results. LASSO penalty selection and all fitted preprocessing used only inner training patients. A sensitivity analysis retained the earliest eligible specimen per patient. Temporal models trained on 351 specimens collected before 1 March 2024; their inner tuning also grouped by patient. Of 134 later specimens, 20 from 16 training patients were excluded from the principal temporal test, leaving 114 specimens from previously unseen patients. All six comparators were evaluated on this identical subset; the full 134-specimen time split is reported separately as an overlapping-patient sensitivity analysis. Patient-cluster bootstrap intervals used 2,000 draws, preserving all specimens and cluster multiplicities and pairing model/baseline draws. The repeated-CV bootstrap recomputed each repeat-level AUC and its mean. The intervals are conditional on stored predictions, without refitting development models, and do not capture the full uncertainty of repeating model development. No external cohort was available. The archived scripts fix 1 March 2024 as the calendar cut-off but do not document its rationale, whether it was chosen before inspection of temporal performance, or whether other dates had previously been explored. We retained this inherited cut-off during revision without searching alternative dates. Temporal findings are therefore treated as exploratory internal estimates, with potential optimism from undocumented cut-off selection unresolved.

Patient-cluster bootstrap intervals use 2,000 draws of patient clusters with replacement. All observations from sampled patients remain together, with cluster multiplicity preserved. Paired AUC differences use the same draws for each model and bacterial count. For the five-repeat summary, each draw recomputes all five pooled AUCs and their mean. These are conditional intervals from fixed saved predictions: models are not refitted in each bootstrap draw. Threshold intervals hold the chosen threshold fixed. Gram intervals for coverage, definite-call accuracy and overall correct yield also resample patients. For the paired accuracy difference, the analyzer and an always-Gram-negative comparator were evaluated on the same 153 definite-result specimens from 131 patients. We resampled these 131 patients with replacement 2,000 times (seed 20260911), retaining all their definite-result specimens and sampled multiplicities. Each draw yielded the specimen-weighted accuracy difference (analyzer minus comparator); its 2.5th and 97.5th percentiles formed the 95% interval. Wilson intervals for definite-call class-specific sensitivity/specificity are provided descriptively and do not adjust patient dependence. Calibration-in-the-large, joint intercept/slope and Brier intervals use 2,000 cluster draws from first-repeat and temporal predictions; Table S11 reports valid draws. NIT/LE and the UTI flag are discrimination comparators only; probability calibration and decision analyses are reported for the three multivariable models. Figure 3D displays quantile-bin counts and a prediction rug; Table S12 gives the underlying bins. No temporal outcomes were used to retune the models.

Four post hoc label sensitivities used patient-grouped outer and inner resampling: exclusion of six yeast-only positives; exclusion of 19 two-organism positives; and assignment of all 20 organism-text-rule likely-contamination reports to either negative or positive. The latter two are boundary scenarios, not claims that these reports were clinically negative or infected. Original primary labels were preserved.

## Computation and model specification

The full 485-specimen reporting model used the same LASSO grid with inner patient-grouped five-fold validation and was not used to estimate validation AUC. Let zⱼ=(xⱼ−meanⱼ)/scaleⱼ after the listed transformation and median imputation. Its predicted probability is p=1/[1+exp(−η)], where η=intercept+Σβⱼzⱼ (Equation S1; logistic model formulation: Hosmer et al., 2013, cited in the main manuscript). Full numerical precision is provided in the code package, together with a standard-library Python predictor numerically checked against the fitted pipeline on all 485 primary specimens (maximum absolute difference below 10⁻¹²). This is a research model specification, not a validated device for withholding culture.

Study protocol, registration and public involvement

A study protocol was submitted with the hospital ethics application. The study was not publicly registered. Patients and the public were not involved in the study design, conduct or dissemination.

## Analysis provenance

Archived R and Python implementations were reconciled during revision, and all reported model results were regenerated using the pipeline specified above. Original files and report-based outcome labels were preserved. CFU/mL-unit parsing was corrected before organism tokenization. A later source audit identified two culture-table records with displaced demographic and analyzer fields. Each was uniquely matched to the report export by specimen identifier, with concordant name and normalized date. Common fields were restored by header in a derived copy, while original specimen/date and culture-specific fields were retained. This recovered one positive and one negative specimen, giving 621 UF/culture pairs and 485 primary specimens; patient groups were rebuilt from restored identifiers. All dependent analyses, intervals and figures were regenerated. Round-trip CSV parsing is retained to preserve floating-point predictions. Additional label sensitivity analyses are explicitly post hoc.

# Supplementary Tables

## Table S1. Organism spectrum in 236 positive specimens

| **Organism or report group** | **Isolates** |
| --- | --- |
| E. coli | 143 |
| E. faecalis | 20 |
| K. pneumoniae | 17 |
| S. agalactiae | 8 |
| A. baumannii report group | 7 |
| S. haemolyticus | 7 |
| Citrobacter spp. | 5 |
| P. mirabilis | 5 |
| E. cloacae | 4 |
| E. faecium | 4 |
| P. aeruginosa | 4 |
| S. epidermidis | 4 |
| S. marcescens | 4 |
| C. albicans | 3 |
| S. gallolyticus subsp. pasteurianus | 3 |
| Other reported organisms | 17 |
| Total | 255 |

Leading groups are shown; Klebsiella pneumoniae includes abbreviated and subspecies-level reports, the A. baumannii report group combines 3 species-level and 4 complex-level reports without upgrading the latter to species identification, and Citrobacter spp. combines reported species. All isolates from retained two-organism positive cultures are counted. Counts describe reports, not independent infected patients. A species that triggers exclusion in a single-organism report can still occur in a retained two-organism report because the archived precedence applies the organism-text rule only to single-organism reports. Full aggregate report-group counts are supplied in pathogen_counts.csv.

## Table S2. Hypothetical culture deferral and missed positive reports

| **Analysis** | **Threshold** | **Deferred** | **Missed positives** | **NPV (95% CI*)** | **Sensitivity (95% CI*)** |
| --- | --- | --- | --- | --- | --- |
| First grouped repeat | <0.05 | 7/485 (1.4%) | 1 | 0.857 | 0.996 |
| First grouped repeat | <0.10 | 66/485 (13.6%) | 4 | 0.939 (0.878–0.987) | 0.983 (0.964–0.996) |
| First grouped repeat | <0.15 | 128/485 (26.4%) | 9 | 0.930 (0.883–0.969) | 0.962 (0.934–0.984) |
| First grouped repeat | <0.20 | 158/485 (32.6%) | 16 | 0.899 | 0.932 |
| First grouped repeat | <0.25 | 185/485 (38.1%) | 22 | 0.881 | 0.907 |
| First grouped repeat | <0.30 | 208/485 (42.9%) | 29 | 0.861 | 0.877 |
| First grouped repeat | <0.40 | 244/485 (50.3%) | 41 | 0.832 | 0.826 |
| First grouped repeat | <0.50 | 268/485 (55.3%) | 52 | 0.806 | 0.780 |
| First grouped repeat; BACT | <10 particles/μL | 107/485 (22.1%) | 7 | 0.935 (0.882–0.976) | 0.970 (0.946–0.990) |
| Patient-disjoint temporal | <0.05 | 1/114 (0.9%) | 0 | 1.000 | 1.000 |
| Patient-disjoint temporal | <0.10 | 15/114 (13.2%) | 1 | 0.933 (0.812–1.000) | 0.982 (0.941–1.000) |
| Patient-disjoint temporal | <0.15 | 33/114 (28.9%) | 1 | 0.970 (0.909–1.000) | 0.982 (0.941–1.000) |
| Patient-disjoint temporal | <0.20 | 43/114 (37.7%) | 2 | 0.953 | 0.964 |
| Patient-disjoint temporal | <0.25 | 48/114 (42.1%) | 7 | 0.854 | 0.873 |
| Patient-disjoint temporal | <0.30 | 52/114 (45.6%) | 8 | 0.846 | 0.855 |
| Patient-disjoint temporal | <0.40 | 59/114 (51.8%) | 10 | 0.831 | 0.818 |
| Patient-disjoint temporal | <0.50 | 65/114 (57.0%) | 14 | 0.785 | 0.745 |
| Patient-disjoint temporal; BACT | <10 particles/μL | 28/114 (24.6%) | 1 | 0.964 (0.895–1.000) | 0.982 (0.939–1.000) |

Patient-cluster 95% intervals are shown for the principal illustrative thresholds (0.10/0.15) and BACT <10; other thresholds are descriptive. Primary N=485, events=236; patient-disjoint temporal N=114, events=55. All estimates condition on report-based exclusion of likely contamination. No cultures were canceled.

## Table S3. Full-cohort LASSO parameters

| **Encoded predictor** | **β** | **Median** | **Mean** | **Scale** |
| --- | --- | --- | --- | --- |
| BACT_log | 1.562491 | 5.722604 | 6.153912 | 3.821291 |
| WBC_log | 0.443024 | 4.030695 | 4.137273 | 2.514873 |
| RBC_log | -0.530488 | 2.240710 | 2.951102 | 2.293010 |
| EC_log | -0.239296 | 1.568616 | 1.768950 | 1.041895 |
| COND_log | 0.000000 | 2.580217 | 2.532099 | 0.398905 |
| NIT | 0.166322 | 0.000000 | 0.313402 | 0.463876 |
| LEU | 0.000000 | 1.000000 | 1.173196 | 1.291887 |
| BLD | 0.315238 | 0.500000 | 1.121649 | 1.238824 |
| PRO | 0.053210 | 0.000000 | 0.563918 | 0.791734 |
| PH | 0.000000 | 6.000000 | 6.109278 | 0.768193 |
| SG | 0.000000 | 1.013000 | 1.014093 | 0.007432 |
| CLOUD | -0.143108 | 0.000000 | 0.323711 | 0.619573 |
| WBC_CLUMPS | 0.177864 | 0.000000 | 0.379381 | 0.485233 |
| UTI_flag | 0.131645 | 0.000000 | 0.371134 | 0.483108 |
| BACT_gram_neg | 0.025204 | 0.000000 | 0.348454 | 0.476481 |
| BACT_gram_pos | -0.095049 | 0.000000 | 0.181443 | 0.385385 |
| age | 0.278503 | 60.000000 | 59.354639 | 13.347458 |
| sex_male | -0.011121 | 0.000000 | 0.393814 | 0.488595 |

Standardized intercept=0.044671036; selected C=0.615848211. The 14 nonzero coefficients are from a full-cohort reporting fit with inner patient-grouped CV, not a validation fit. Medians/means/scales apply to encoded transformed inputs. Full-precision parameters and Equation S1 are supplied; selection may vary across training partitions.

## Table S4. Discrimination across internal validation analyses

| **Analysis / estimand** | **Model** | **N /events** | **AUC (95% CI)** |
| --- | --- | --- | --- |
| Mean of 5 grouped-repeat AUCs | Bacterial count | 485 /236 | 0.892 (0.864–0.919) |
| Mean of 5 grouped-repeat AUCs | Gradient boosting | 485 /236 | 0.893 (0.865–0.919) |
| Mean of 5 grouped-repeat AUCs | LASSO | 485 /236 | 0.895 (0.866–0.921) |
| Mean of 5 grouped-repeat AUCs | Nitrite + LE | 485 /236 | 0.838 (0.801–0.873) |
| Mean of 5 grouped-repeat AUCs | Random forest | 485 /236 | 0.897 (0.869–0.924) |
| Mean of 5 grouped-repeat AUCs | Analyzer UTI flag | 485 /236 | 0.799 (0.765–0.832) |
| First grouped repeat | Bacterial count | 485 /236 | 0.892 (0.863–0.918) |
| First grouped repeat | Gradient boosting | 485 /236 | 0.895 (0.865–0.923) |
| First grouped repeat | LASSO | 485 /236 | 0.892 (0.865–0.919) |
| First grouped repeat | Random forest | 485 /236 | 0.895 (0.867–0.923) |
| First grouped repeat | Nitrite + LE | 485 /236 | 0.836 (0.801–0.873) |
| First grouped repeat | Analyzer UTI flag | 485 /236 | 0.799 (0.766–0.832) |
| Earliest specimen/patient | Bacterial count | 377 /186 | 0.896 (0.863–0.927) |
| Earliest specimen/patient | LASSO | 377 /186 | 0.879 (0.841–0.914) |
| Patient-disjoint temporal | Bacterial count | 114 /55 | 0.927 (0.877–0.965) |
| Patient-disjoint temporal | Gradient boosting | 114 /55 | 0.908 (0.850–0.956) |
| Patient-disjoint temporal | LASSO | 114 /55 | 0.919 (0.860–0.963) |
| Patient-disjoint temporal | Random forest | 114 /55 | 0.901 (0.836–0.952) |
| Patient-disjoint temporal | Nitrite + LE | 114 /55 | 0.883 (0.818–0.941) |
| Patient-disjoint temporal | Analyzer UTI flag | 114 /55 | 0.802 (0.725–0.872) |
| Full time split; patient overlap | Bacterial count | 134 /65 | 0.935 (0.895–0.969) |
| Full time split; patient overlap | Gradient boosting | 134 /65 | 0.911 (0.854–0.957) |
| Full time split; patient overlap | LASSO | 134 /65 | 0.925 (0.876–0.964) |
| Full time split; patient overlap | Random forest | 134 /65 | 0.906 (0.849–0.953) |
| Full time split; patient overlap | Nitrite + LE | 134 /65 | 0.880 (0.817–0.937) |
| Full time split; patient overlap | Analyzer UTI flag | 134 /65 | 0.817 (0.752–0.880) |

Intervals are conditional patient-cluster percentiles (2,000 draws). Every primary outer and inner split groups by patient. The first grouped repeat is part of, not independent from, the five-repeat summary. Full-time-split results include patient overlap and are supplemental only. The earliest-specimen sensitivity fits LASSO and the BACT baseline; other models were not evaluated in that analysis. LE, leukocyte esterase.

Paired mean-repeat ΔAUC versus bacterial count: Gradient boosting +0.0012 (95% CI -0.0157 to +0.0185); LASSO +0.0027 (95% CI -0.0077 to +0.0131); Nitrite + LE -0.0541 (95% CI -0.0839 to -0.0256); Random forest +0.0053 (95% CI -0.0081 to +0.0192); Analyzer UTI flag -0.0931 (95% CI -0.1208 to -0.0654). These intervals do not establish equivalence.

## Table S5. Exploratory contamination classification

| **Threshold** | **TP / FP / TN / FN** | **Sensitivity (CI)** | **Specificity (CI)** | **Precision (CI)** | **F1 (CI)** |
| --- | --- | --- | --- | --- | --- |
| ≥0.10 | 130/431/54/6 | 0.956 (0.916–0.986) | 0.111 (0.083–0.142) | 0.232 (0.194–0.269) | 0.373 (0.324–0.420) |
| ≥0.20 | 115/280/205/21 | 0.846 (0.780–0.905) | 0.423 (0.377–0.469) | 0.291 (0.243–0.338) | 0.433 (0.374–0.485) |
| ≥0.30 | 51/72/413/85 | 0.375 (0.289–0.460) | 0.852 (0.818–0.884) | 0.415 (0.321–0.505) | 0.394 (0.309–0.469) |
| ≥0.50 | 8/9/476/128 | 0.059 (0.022–0.101) | 0.981 (0.969–0.992) | 0.471 (0.222–0.706) | 0.105 (0.041–0.175) |

Patient-grouped predictions; 621 specimens, 136 events. AUC 0.701 (95% CI 0.647–0.753), average precision 0.367, event fraction 0.219, Brier 0.159, joint calibration intercept -0.034 and slope 1.047. Squamous epithelial count AUC 0.640 (95% CI 0.588–0.693). No class balancing was applied. The model is exploratory; no threshold is recommended for specimen discard or recollection.

## Table S6. Post hoc label-definition sensitivity analyses

| **Scenario** | **N /positives** | **LASSO AUC (CI)** | **BACT AUC (CI)** |
| --- | --- | --- | --- |
| Exclude yeast-only | 479 /230 | 0.899 (95% CI 0.871–0.926) | 0.895 (95% CI 0.866–0.921) |
| Exclude two-organism | 466 /217 | 0.897 (95% CI 0.865–0.923) | 0.894 (95% CI 0.864–0.921) |
| Organism-text reports → negative | 505 /236 | 0.883 (95% CI 0.852–0.911) | 0.883 (95% CI 0.854–0.909) |
| Organism-text reports → positive | 505 /256 | 0.883 (95% CI 0.853–0.911) | 0.883 (95% CI 0.852–0.910) |

Each uses patient-grouped outer and inner resampling, 2,000 patient-cluster conditional intervals, and corrected CFU-unit parsing. These sensitivity rules assess dependence on study labels and do not adjudicate infection or prove that presumed contamination is negative.

## Table S7. Measured computational cost

| **Model** | **Outer fits** | **Median training seconds/fit** | **Median batch inference ms/specimen** |
| --- | --- | --- | --- |
| Gradient boosting | 50 | 0.0923 | 0.0682 |
| LASSO | 50 | 0.4076 | 0.0235 |
| Nitrite + LE | 50 | 0.0032 | 0.0195 |
| Random forest | 50 | 0.5998 | 1.8391 |

Primary fits on Intel(R) Core(TM) Ultra 9 185H, Linux-6.18.33.2-microsoft-standard-WSL2-x86_64-with-glibc2.39, Python 3.12.3, scikit-learn 1.9.0; two workers. Training samples range 434–438; test samples 47–51. LASSO fit times include inner search/refit; RF/GB use fixed settings. Inference divides batch time by test size and includes pipeline overhead. Full-cohort LASSO reporting fit: 2.236 s; median single-specimen inference over 100 runs: 1.089 ms; serialized pipeline: 2749 bytes. These are implementation measurements, not smart-device latency, energy, safety or external validation.

## Table S8. Gram classification denominators and performance

| **Measure** | **Numerator /denominator** | **Estimate** | **Uncertainty /comparator** |
| --- | --- | --- | --- |
| Definite-result coverage | 153/211 | 72.5% | 66.3–78.5% (patient cluster) |
| Accuracy when definite | 144/153 | 94.1% | 90.2–97.4% (patient cluster) |
| Always-Gram-negative accuracy, same subset | 131/153 | 85.6% | 79.2–90.3% (Wilson, descriptive) |
| Paired accuracy difference, definite subset | (144 − 131)/153 | 8.5 percentage points | 2.0–15.3 percentage points (paired patient cluster) |
| Correct definite result among all eligible | 144/211 | 68.2% | 61.9–74.5% (patient cluster) |
| Gram-negative sensitivity, definite subset | 124/131 | 94.7% | 89.4–97.4% (Wilson, descriptive) |
| Gram-negative specificity, definite subset | 20/22 | 90.9% | 72.2–97.5% (Wilson, descriptive) |
| Balanced accuracy, definite subset | Mean of two class recalls | 92.8% | Majority-class baseline 50.0% |
| Cohen κ, definite subset | 153 definite calls | 0.782 | Descriptive point estimate |
| Gram-negative definite coverage | 131/175 | 74.9% | 67.9–81.6% (patient cluster) |
| Gram-negative correct definite/all class | 124/175 | 70.9% | 63.8–77.9% (patient cluster) |
| Gram-positive definite coverage | 22/36 | 61.1% | 44.1–77.1% (patient cluster) |
| Gram-positive correct definite/all class | 20/36 | 55.6% | 38.9–70.6% (patient cluster) |

Culture truth: 175 Gram-negative and 36 Gram-positive reports. Definite-call subset: 131 Gram-negative and 22 Gram-positive. Of 58 non-definite reports, 17 had dual Gram-positive/Gram-negative flags, 36 had no BACT Info alert and five were unclassified. Confusion matrix [GN→GN, GN→GP; GP→GN, GP→GP]=[124,7;2,20]. Wilson intervals assume independent specimens and are descriptive; principal coverage/accuracy/yield intervals use patient clustering. The paired difference uses the same definite-result specimens for both classifiers: 20 specimens were correct only for the analyzer and seven only for the comparator. Its patient-cluster interval conditions on a definite result and does not estimate an all-specimen or clinical benefit. A hypothetical always-Gram-negative classifier on all 211 would have 175 correct (82.9%) but no ability to recognize Gram-positive organisms; this is not directly the same performance measure as correct definitive-result yield with incomplete recorded classification. Gram-negative specimens comprised 124 correct, 7 wrong, 17 dual-flag results, 25 with no BACT Info alert and two unclassified results; Gram-positive specimens comprised 20 correct, 2 wrong, no dual-flag results, 11 with no BACT Info alert and three unclassified results. Class-specific all-eligible yields include the cost of dual-flag, unclassified and no-alert results; they are not conditional class sensitivity. The laboratory confirmed that the report descriptors distinguish absence of a BACT Info alert from an indeterminate scattergram classification (Unclassified). Both categories lack a definite Gram classification; neither represents a missing exported value. The 211 eligible specimens represent 173 patients; 153 definite specimens represent 131 patients. The definite Gram-negative and Gram-positive subsets represent 112 and 20 patients, respectively. These patient counts can overlap between culture classes. The 144/211 yield is conditional on the post-culture-defined subset and is not an all-incoming-specimen workflow yield.

## Table S9. Actual Table 1 data availability and percentage denominators

| **Characteristic** | **Not cultured: available / missing** | **Cultured: available / missing** | **Percentage denominator: not cultured / cultured** | **Definition** |
| --- | --- | --- | --- | --- |
| Bacterial count, particles/µL | 31,223 / 6,441 | 621 / 22 | — | available numeric observations |
| White-blood-cell count, /µL | 31,223 / 6,441 | 621 / 22 | — | available numeric observations |
| Red-blood-cell count, /µL | 31,220 / 6,444 | 619 / 24 | — | available numeric observations |
| Epithelial-cell count, /µL | 31,223 / 6,441 | 621 / 22 | — | available numeric observations |
| Squamous epithelial cells, /µL | 31,223 / 6,441 | 621 / 22 | — | available numeric observations |
| Transitional epithelial cells, /µL | 31,223 / 6,441 | 621 / 22 | — | available numeric observations |
| Conductivity, mS/cm | 31,223 / 6,441 | 621 / 22 | — | available numeric observations |
| pH | 37,612 / 52 | 643 / 0 | — | available numeric observations |
| Specific gravity | 37,601 / 63 | 643 / 0 | — | available numeric observations |
| Age, years | 33,424 / 4,240 | 643 / 0 | — | available numeric observations |
| Nitrite | 37,612 / 52 | 643 / 0 | 37,612 / 643 | available binary results |
| Leukocyte esterase | 37,612 / 52 | 643 / 0 | 37,664 / 643 | total group; ordinal >=1 |
| Blood | 37,612 / 52 | 643 / 0 | 37,664 / 643 | total group; ordinal >=1 |
| Protein | 37,612 / 52 | 643 / 0 | 37,664 / 643 | total group; ordinal >=1 |
| Turbidity (cloudy) | 37,601 / 63 | 643 / 0 | 37,664 / 643 | total group; cloudy |
| Analyser UTI flag | All records encoded; measurement availability unknown | All records encoded; measurement availability unknown | 37,664 / 643 | Recorded target text; absent/blank text = 0 |
| WBC clumps | 31,223 / 6,441 | 621 / 22 | 31,223 / 621 | available binary results |
| Presumptive Gram-negative flag | All records encoded; measurement availability unknown | All records encoded; measurement availability unknown | 37,664 / 643 | Recorded target text; absent/blank text = 0 |
| Presumptive Gram-positive flag | All records encoded; measurement availability unknown | All records encoded; measurement availability unknown | 37,664 / 643 | Recorded target text; absent/blank text = 0 |
| Male sex | 33,422 / 4,242 | 643 / 0 | 33,422 / 643 | recognized sex |
| Female sex | 33,422 / 4,242 | 643 / 0 | 33,422 / 643 | recognized sex |

Total groups comprise 37,664 not-cultured and 643 cultured specimens. Available/missing counts refer to the stated numeric or categorical encoding; they are not model-imputation counts. Continuous summaries use available numeric observations and have no percentage denominator. Binary nitrite and WBC-clump percentages use available binary results; sex percentages use recognized male/female results. Ordinal positivity (≥1), cloudy appearance and analyzer text flags retain the original total-group denominator. For analyzer flags, absence of the target text is encoded as 0, including blank source text; the resulting available count therefore does not establish that a measured negative flag was returned for every specimen. Table 1 contains unadjusted descriptive comparisons, not an imputed analysis. The two restored records are included in the cultured numeric counts. Flag proportions describe recorded target-text presence among all workflow specimens; encoded completeness does not establish complete measurements or negative measured results. Their SMDs combine reporting availability and flag occurrence.

## Table S10. Exploratory contamination predictor availability

| **Encoded predictor** | **Available /621** | **Missing /621 (%)** |
| --- | --- | --- |
| SQUA_EC | 621 | 0 (0.0%) |
| EC | 621 | 0 (0.0%) |
| TRAN_EC | 621 | 0 (0.0%) |
| BACT | 621 | 0 (0.0%) |
| WBC | 621 | 0 (0.0%) |
| BACT_gram_neg | 621 | 0 (0.0%) |
| BACT_gram_pos | 621 | 0 (0.0%) |
| CLOUD | 621 | 0 (0.0%) |
| COLOR_dark | 603 | 18 (2.9%) |
| age | 621 | 0 (0.0%) |
| sex_male | 621 | 0 (0.0%) |

Availability is assessed before model imputation after archived field encoding. Text-flag zeros include absent/blank descriptors and do not establish measured negative results. COLOR_dark is the ordinal color field despite its historical name; 18 missing values were imputed within training partitions only.

## Table S11. Calibration uncertainty

| **Analysis** | **Model** | **Metric** | **Estimate (95% CI)** | **Valid /2000** |
| --- | --- | --- | --- | --- |
| First grouped repeat | LASSO | Calibration-in-the-large | 0.026 (-0.214–0.262) | 2000 |
| First grouped repeat | LASSO | Joint intercept | 0.033 (-0.212–0.289) | 2000 |
| First grouped repeat | LASSO | Joint slope | 1.032 (0.878–1.229) | 2000 |
| First grouped repeat | LASSO | Brier score | 0.129 (0.111–0.147) | 2000 |
| First grouped repeat | Random forest | Calibration-in-the-large | -0.008 (-0.235–0.217) | 2000 |
| First grouped repeat | Random forest | Joint intercept | 0.006 (-0.239–0.260) | 2000 |
| First grouped repeat | Random forest | Joint slope | 1.085 (0.936–1.281) | 2000 |
| First grouped repeat | Random forest | Brier score | 0.126 (0.109–0.144) | 2000 |
| First grouped repeat | Gradient boosting | Calibration-in-the-large | -0.070 (-0.398–0.276) | 2000 |
| First grouped repeat | Gradient boosting | Joint intercept | -0.077 (-0.316–0.163) | 2000 |
| First grouped repeat | Gradient boosting | Joint slope | 0.684 (0.577–0.837) | 2000 |
| First grouped repeat | Gradient boosting | Brier score | 0.127 (0.105–0.150) | 2000 |
| Disjoint temporal | LASSO | Calibration-in-the-large | 0.006 (-0.523–0.514) | 2000 |
| Disjoint temporal | LASSO | Joint intercept | 0.028 (-0.560–0.699) | 2000 |
| Disjoint temporal | LASSO | Joint slope | 1.081 (0.825–1.537) | 2000 |
| Disjoint temporal | LASSO | Brier score | 0.120 (0.082–0.163) | 2000 |
| Disjoint temporal | Random forest | Calibration-in-the-large | -0.016 (-0.495–0.463) | 2000 |
| Disjoint temporal | Random forest | Joint intercept | -0.007 (-0.555–0.542) | 2000 |
| Disjoint temporal | Random forest | Joint slope | 1.071 (0.788–1.535) | 2000 |
| Disjoint temporal | Random forest | Brier score | 0.126 (0.086–0.167) | 2000 |
| Disjoint temporal | Gradient boosting | Calibration-in-the-large | -0.038 (-0.766–0.651) | 2000 |
| Disjoint temporal | Gradient boosting | Joint intercept | -0.035 (-0.618–0.501) | 2000 |
| Disjoint temporal | Gradient boosting | Joint slope | 0.702 (0.504–1.082) | 2000 |
| Disjoint temporal | Gradient boosting | Brier score | 0.125 (0.081–0.173) | 2000 |

Percentile intervals resample patient clusters from saved first-repeat or temporal predictions. Calibration-in-the-large uses an offset slope of one; joint intercept and slope are estimated together. These quantify conditional sampling uncertainty, not uncertainty from refitting the complete development process. No recalibration is applied to the predictions used to calculate AUC, Brier, thresholds or decision curves.

## Table S12. First-repeat grouped LASSO calibration bins

| **Probability range** | **N /events** | **Mean predicted** | **Observed proportion** |
| --- | --- | --- | --- |
| 0.0304–0.0858 | 49 /3 | 0.067 | 0.061 |
| 0.0866–0.1164 | 49 /4 | 0.103 | 0.082 |
| 0.1172–0.1834 | 48 /3 | 0.147 | 0.062 |
| 0.1870–0.2659 | 48 /14 | 0.223 | 0.292 |
| 0.2670–0.3993 | 49 /17 | 0.327 | 0.347 |
| 0.3999–0.6077 | 48 /27 | 0.497 | 0.562 |
| 0.6096–0.8113 | 48 /34 | 0.720 | 0.708 |
| 0.8126–0.8887 | 49 /43 | 0.860 | 0.878 |
| 0.8911–0.9487 | 48 /45 | 0.924 | 0.938 |
| 0.9498–0.9850 | 49 /46 | 0.965 | 0.939 |

Bins use prediction quantiles, with coincident quantiles merged. The bins are descriptive; metric-level patient-cluster intervals are in Table S11.

## Table S13. Archived organism-text tokens and explanatory names

| **Rule ID** | **English description / Latin name** | **Matching mode** |
| --- | --- | --- |
| T01 | *Staphylococcus epidermidis* | Substring in a single-organism report, subject to rule precedence |
| T02 | *Corynebacterium striatum* | Substring in a single-organism report, subject to rule precedence |
| T03 | *Staphylococcus lugdunensis* | Substring in a single-organism report, subject to rule precedence |
| T04 | *Lactobacillus iners* | Substring in a single-organism report, subject to rule precedence |
| T05 | *Ligilactobacillus salivarius (formerly Lactobacillus salivarius)* | Substring in a single-organism report, subject to rule precedence |
| T06 | *Gardnerella vaginalis* | Substring in a single-organism report, subject to rule precedence |
| T07 | *Lactobacillus acidophilus* | Substring in a single-organism report, subject to rule precedence |
| T08 | *Corynebacterium glucuronolyticum* | Substring in a single-organism report, subject to rule precedence |
| T09 | *Corynebacterium simulans* | Substring in a single-organism report, subject to rule precedence |
| T10 | *Corynebacterium tuberculostearicum* | Substring in a single-organism report, subject to rule precedence |
| T11 | *Corynebacterium kroppenstedtii* | Substring in a single-organism report, subject to rule precedence |
| T12 | Generic Corynebacterium-related text; no species resolution | Substring in a single-organism report, subject to rule precedence |
| T13 | *Streptococcus oralis* | Exact single-organism string |
| T14 | *Streptococcus mitis* | Exact single-organism string |

Table S13 provides English descriptions of the archived matching terms; it does not reidentify isolates. The original Chinese strings and rule implementation remain in the archived analysis code. The generic corynebacterial substring is not equivalent to excluding every organism in a taxonomic genus. The investigators confirmed that T09 corresponds to Corynebacterium simulans and T11 to Corynebacterium kroppenstedtii. These clarifications update the English names only; the archived matching rules and outcome labels remain unchanged. Ligilactobacillus salivarius is the current name of Lactobacillus salivarius.

## Table S14. Archived independent-specimen test calculations (audit only)

| **Characteristic** | **Test** | **N: not cultured / cultured** | **Statistic** | **df** | **Two-sided P** |
| --- | --- | --- | --- | --- | --- |
| Bacterial count, particles/µL | U | 31,223 / 621 | U = 5,154,288.5; z = -20.023339 | — | 3.448221e-89 |
| White-blood-cell count, /µL | U | 31,223 / 621 | U = 4,518,274.0; z = -22.822580 | — | 2.7367213e-115 |
| Red-blood-cell count, /µL | U | 31,220 / 619 | U = 6,251,410.0; z = -15.066538 | — | 2.6883784e-51 |
| Epithelial-cell count, /µL | U | 31,223 / 621 | U = 7,011,814.5; z = -11.830000 | — | 2.7314325e-32 |
| Squamous epithelial cells, /µL | U | 31,223 / 621 | U = 7,892,877.5; z = -7.966619 | — | 1.6307502e-15 |
| Transitional epithelial cells, /µL | U | 31,223 / 621 | U = 6,741,615.0; z = -14.280270 | — | 2.9047537e-46 |
| Conductivity, mS/cm | U | 31,223 / 621 | U = 11,606,452.5; z = 8.427834 | — | 3.5212157e-17 |
| pH | U | 37,612 / 643 | U = 11,360,727.0; z = -2.70325876968005 | — | 0.0068663267963244 |
| Specific gravity | U | 37,601 / 643 | U = 14,087,511.5; z = 7.207007 | — | 5.7195006e-13 |
| Age, years | U | 33,424 / 643 | U = 8,272,299.0; z = -10.016248 | — | 1.2931926e-23 |
| Nitrite | χ² | 37,612 / 643 | χ² = 1156.994235 | 1 | 1.3545468e-253 |
| Leukocyte esterase | χ² | 37,664 / 643 | χ² = 719.782423 | 1 | 1.4926943e-158 |
| Blood | χ² | 37,664 / 643 | χ² = 839.686459 | 1 | 1.2698954e-184 |
| Protein | χ² | 37,664 / 643 | χ² = 570.201844 | 1 | 5.0746972e-126 |
| Turbidity (cloudy) | χ² | 37,664 / 643 | χ² = 754.016407 | 1 | 5.3714697e-166 |
| Analyser UTI flag | χ² | 37,664 / 643 | χ² = 1333.640298 | 1 | 5.530756e-292 |
| WBC clumps | χ² | 31,223 / 621 | χ² = 1120.806801 | 1 | 9.9239294e-246 |
| Presumptive Gram-negative flag | χ² | 37,664 / 643 | χ² = 1124.001411 | 1 | 2.0061617e-246 |
| Presumptive Gram-positive flag | χ² | 37,664 / 643 | χ² = 157.553346 | 1 | 3.8746969e-36 |
| Male sex | χ² | 33,422 / 643 | χ² = 19.635742 | 1 | 9.3699908e-06 |
| Female sex | χ² | 33,422 / 643 | χ² = 19.635742 | 1 | 9.3699908e-06 |

U is oriented to the non-cultured group and uses average ranks for ties. The reported normal z uses tie-corrected variance and a 0.5 continuity correction toward the null. Pearson χ² uses no continuity correction; all categorical tests have one degree of freedom. Counts match the archived variable-specific encoding in Table S9, including full-group denominators for selected ordinal/text flags. These calculations retain the independent-specimen assumption, which is not met for repeated patients; they have no patient-adjusted inferential interpretation, are excluded from the main Table 1, and do not support group-difference conclusions. More precise statistics and counts are supplied in aggregate/table1_test_statistics.csv with the code. Female and male rows describe the same complementary comparison. Displayed statistics may be rounded; P values were calculated at full precision. The pH row retains additional digits to make its statistic–P relationship directly reproducible.

## Table S15. Temporal training and patient-disjoint test characteristics

| **Encoded field** | **Training: summary** | **Available / missing** | **Test: summary** | **Available / missing** |
| --- | --- | --- | --- | --- |
| age | 60 (53–68) | 351 / 0 | 60 (48–68.75) | 114 / 0 |
| sex_male | 137/351 (39.0%) | 351 / 0 | 42/114 (36.8%) | 114 / 0 |
| BACT | 315.5 (13.15–2.667e+04) | 351 / 0 | 223.4 (10.45–3.452e+04) | 114 / 0 |
| WBC | 52.4 (5.45–382.5) | 351 / 0 | 70.05 (6.1–305.3) | 114 / 0 |
| RBC | 8.75 (2.3–83.2) | 350 / 1 | 7.9 (2.8–31.8) | 113 / 1 |
| EC | 3.6 (1.6–9.5) | 351 / 0 | 4.85 (1.85–10.52) | 114 / 0 |
| COND | 12.3 (8.65–15.85) | 351 / 0 | 11.9 (9.3–14.57) | 114 / 0 |
| NIT | 112/351 (31.9%) | 351 / 0 | 34/114 (29.8%) | 114 / 0 |
| LEU | 0 (0–3) | 351 / 0 | 1 (0–2) | 114 / 0 |
| BLD | 0.5 (0–3) | 351 / 0 | 0.5 (0–2) | 114 / 0 |
| PRO | 0 (0–1) | 351 / 0 | 0 (0–1) | 114 / 0 |
| PH | 6 (5.5–6.5) | 351 / 0 | 6 (5.5–6.5) | 114 / 0 |
| SG | 1.013 (1.009–1.018) | 351 / 0 | 1.013 (1.008–1.017) | 114 / 0 |
| CLOUD | 0 (0–1) | 351 / 0 | 0 (0–0) | 114 / 0 |
| WBC_CLUMPS | 135/351 (38.5%) | 351 / 0 | 43/114 (37.7%) | 114 / 0 |
| UTI_flag | 131/351 (37.3%) | 351 / 0 | 41/114 (36.0%) | 114 / 0 |
| BACT_gram_neg | 119/351 (33.9%) | 351 / 0 | 43/114 (37.7%) | 114 / 0 |
| BACT_gram_pos | 67/351 (19.1%) | 351 / 0 | 19/114 (16.7%) | 114 / 0 |

Training: 351 specimens from 276 patients before 1 March 2024, including 171 positive reports (48.7%). Test: 114 later specimens from 101 patients absent from training, including 55 positive reports (48.2%). Values are specimen-weighted median (25th–75th percentile) on the encoded pre-log scale, or positive count / available count (%). Predictor definitions and units are in the dictionary above. LEU, BLD, PRO and CLOUD are ordinal encoded values. Recorded flag zeros include absent text, so complete encoded availability does not prove that every flag was clinically measured or displayed. No hypothesis tests were applied; these summaries describe case mix, not a causal explanation of temporal performance.

## Table S16. Numeric boundary and negative-value audit before model transformations

| **Numeric field** | **Boundary n /485** | **Boundary n /621** | **Source boundary token** | **Negative n /485** | **Negative n /621** |
| --- | --- | --- | --- | --- | --- |
| BACT | 53 | 54 | >100000 | 0 | 0 |
| WBC | 0 | 0 | None | 0 | 0 |
| RBC | 0 | 0 | None | 0 | 0 |
| EC | 0 | 0 | None | 0 | 0 |
| SQUA_EC | 0 | 0 | None | 0 | 0 |
| TRAN_EC | 0 | 0 | None | 0 | 0 |
| COND | 0 | 0 | None | 0 | 0 |
| PH | 0 | 0 | None | 0 | 0 |
| SG | 0 | 0 | None | 0 | 0 |
| age | 0 | 0 | None | 0 | 0 |

Primary cohort: 485 report-positive/negative specimens. UF cohort: 621 specimens, including likely contamination. Comparator symbols were audited against the source worksheet; missing values were not counted as negative numbers or boundary tokens. The numeric parser retained the reported boundary for inequalities. SQUA_EC and TRAN_EC are used only in the exploratory contamination model. Ordinal and text-flag encodings are described separately in the predictor dictionary. No defensive negative-value truncation changed a value in either cohort. BACT rank bounds and their limited interpretation are given in Supplementary Methods.
