# Supplementary material for: Automated urinalysis for predicting urine culture positivity and presumptive Gram classification: a retrospective study at a cancer hospital
Source: Front Cell Infect Microbiol. 2026 Oct 12;16:1935652. doi: 10.3389/fcimb.2026.1935652 (PMC13696273; doi:10.3389/fcimb.2026.1935652)
Supplement: Supplementary file 2 [file Table1.docx]

TRIPOD+AI Reporting Crosswalk

The 27 main items and 52 subitems follow TRIPOD+AI (2024). Requirement labels are abbreviated paraphrases; the official checklist supplies the full wording. Status describes reporting, not methodological quality or certification. D indicates development; E indicates evaluation. Items not applicable to an internally validated model are distinguished from unavailable information.

Collins GS, Moons KGM, Dhiman P, Riley RD, Beam AL, Van Calster B, et al. TRIPOD+AI statement: updated guidance for reporting clinical prediction models that use regression or machine learning methods. BMJ. 2024;385:e078378. doi:10.1136/bmj-2023-078378.

Official checklist: https://www.tripod-statement.org/wp-content/uploads/2024/04/TRIPODAI-Supplement.pdf

| **Item** | **Requirement** | **Status** | **Location and reporting note** |
| --- | --- | --- | --- |
| 1 | Title identification | Partial | Title The title identifies a retrospective cancer-hospital study and the laboratory endpoint; model development and internal evaluation are described in the Abstract and Methods rather than explicitly in the title. |
| 2 | Abstract reporting | Partial | Abstract Design, cohorts, methods, results, uncertainty and clinical limits are summarized. Model availability, registration and funding are not in abstract; use separate abstract checklist if required. |
| 3a | Clinical rationale | Reported | Introduction Prior urinalysis studies and rationale for laboratory prediction are described. |
| 3b | Intended use/users | Partial | Introduction; Methods 2.3; Discussion Culture-ordered laboratory setting and possible future applications are described. No operational user specification or deployed pathway was tested. |
| 3c | Health inequalities | Not reported | No dedicated section No sourced account of relevant sociodemographic health inequalities is provided. Culture-ordering selection is not a fairness analysis. |
| 4 | Study objectives | Reported | Introduction Development/internal evaluation, simple-model comparison, Gram validation and exploratory contamination objectives are distinguished. |
| 5a | Data provenance | Reported | Methods 2.1; 2.4.3 Routine single-center records underpin development and internal temporal evaluation; representativeness is limited and discussed. |
| 5b | Accrual dates | Reported | Methods 2.1; 2.4.3 1 June 2023–27 June 2024; temporal split at 1 March 2024. No prognostic follow-up endpoint. |
| 6a | Setting | Reported | Methods 2.1; affiliations One tertiary cancer hospital in Shenzhen, China; analyzer platforms specified. |
| 6b | Eligibility | Reported | Methods 2.1–2.3; Figure 1 Specimen linking, UF requirement and task-specific exclusions are described. |
| 6c | Treatments | Unavailable | Methods 2.1; Discussion Antimicrobial exposure and treatment decisions were unavailable; no treatment-adjusted analysis was performed. |
| 7 | Data preparation | Partial | Methods 2.1–2.4; code Deduplication, report parsing, transforms and fold-local preprocessing documented. Group-specific data-quality differences were not assessed. |
| 8a | Endpoint assessment | Reported with limitations | Methods 2.2; Supplementary Methods  Laboratory thresholds and author-confirmed bacterial/two-organism significance are specified; yeast reporting is distinguished from clinical infection. Quantitative counts are not independently recoverable for most positive reports in the export; uniformity across demographic groups was not evaluated. |
| 8b | Outcome assessors | Unavailable | Methods 2.2; Discussion Routine contextual report interpretation is acknowledged; number, qualifications and demographic characteristics of report assessors are not documented. |
| 8c | Outcome blinding | Not established | Methods 2.2; Discussion Routine reports could incorporate urinalysis and clinical information. No blinded reference assessment is demonstrated; incorporation bias is explicitly possible. |
| 9a | Candidate selection | Partial | Methods 2.3; Supplementary Methods Candidate set enumerated as available routine parameters; selection rationale and any prior omissions require the predictor dictionary/code rather than assumptions. |
| 9b | Predictor measurement | Partial | Methods 2.1,2.3; Supplementary Methods Platforms, variables, transforms and coding described. No formal predictor-assessor blinding procedures are evidenced. |
| 9c | Predictor assessors | Not applicable to automated measurements | Methods 2.1,2.3 Primary measured predictors are instrument outputs or recorded demographics; no subjective image/clinical scoring predictor is described. |
| 10 | Sample-size rationale | Reported with limitations | Methods 2.4.2 All available eligible records; post hoc assumptions, patient dependence and contamination shortfall disclosed. This does not certify adequacy. |
| 11 | Missingness handling | Reported | Methods 2.3–2.4; Supplementary Tables S9–S10 Primary RBC missingness, contamination-model color missingness, encoded flag availability and training-only median imputation are described. Complete encoded flag fields are not equated with verified measurement availability. |
| 12a | Data partitioning | Reported | Methods 2.4.1–2.4.3; code All five primary outer repeats and inner tuning splits group by patient. The principal temporal test excludes training patients and uses the same 114 specimens for all six comparators; the full overlapping time split is supplemental. The inherited calendar cut-off is documented; its historical rationale and prespecification status could not be verified, so temporal estimates are exploratory. |
| 12b | Predictor processing | Reported | Methods 2.3,2.4.1; code; Table S16 Truncation, log transforms, imputation and standardization are reproducibly specified. Exact inequality counts and zero negative-value counts are audited; BACT censoring rank bounds are reported. |
| 12c | Model construction | Reported | Methods 2.4.1–2.4.3; code Model families, fixed/tuned hyperparameters, patient-isolated nested primary validation and earliest-specimen sensitivity are specified. |
| 12d | Cluster heterogeneity | Not assessed | Methods 2.1,2.4.3 Single hospital; no between-center heterogeneity analysis. Patient grouping/bootstrap handles dependence, not demographic or center heterogeneity. |
| 12e | Performance methods | Reported | Methods 2.4.3–2.4.4; Figures 3–4 AUC comparisons, calibration, bootstrap uncertainty and conditional decision scenarios specified. |
| 12f | Updating methods | Not applicable: no update | Methods 2.4.4 Calibration was evaluated diagnostically; no externally updated model or recalibration intervention is reported. |
| 12g | Prediction calculation | Reported | Methods 2.4.1; code; Supplementary Table S3 Fold-trained pipeline generated probabilities; fixed comparators used measured scores; model specification is supplied. |
| 13 | Imbalance handling | Reported | Discussion; Supplementary Methods Observed class distribution retained; no automatic sampling correction. Exploratory threshold metrics and rationale given. |
| 14 | Fairness methods | Not assessed | No dedicated analysis No sex-, age- or other demographic fairness assessment or mitigation is reported. Do not infer fairness from overall performance. |
| 15 | Output/thresholds | Reported | Methods; Results Probability outputs and illustrative thresholds are separated from raw bacterial score; prevalence operating point selected retrospectively. |
| 16 | Dataset differences | Reported | Methods 2.4.3; Results 3.3; Tables S4 and S15 Temporal training/test dates, specimen and unique-patient counts, event counts, and predictor distributions and encoded-data availability are reported. Comparisons are descriptive; clinical variables unavailable in the source remain unavailable. The inherited calendar cut-off is documented; its historical rationale and prespecification status could not be verified, so temporal estimates are exploratory. |
| 17 | Ethics/consent | Reported | Ethics Statement The committee, author-specified approval number IIT2026-40-1, consent waiver and ethical protections are stated. Only existing routine laboratory records were analyzed; no additional research testing of residual specimens was performed. |
| 18a | Funding/role | Reported | Funding The funder had no role in design, analysis, writing or the submission decision. |
| 18b | Conflicts | Reported | Conflict of Interest The authors report no potential conflict of interest. |
| 18c | Protocol access | Partial | Supplementary Methods, Study protocol, registration and public involvement  A protocol was submitted with the ethics application. Public access to the protocol is not specified. |
| 18d | Registration | Reported | Supplementary Methods, Study protocol, registration and public involvement  The study was not publicly registered. |
| 18e | Data access | Reported with conditions | Data Availability De-identified data on request subject to institutional requirements; no public repository falsely claimed. |
| 18f | Code access | Reported | Methods 2.4.1 and 2.4.4; Supplementary Material The code, dictionary and fitted parameters accompany the supplementary package; public web hosting is not asserted. |
| 19 | Public involvement | Reported | Supplementary Methods, Study protocol, registration and public involvement  Patients and the public were not involved in study design, conduct or dissemination. |
| 20a | Participant flow | Reported | Results 3.1; Figure 1 Specimen flow and patient totals distinguish 621/477 and primary 485/377; outcome counts provided. |
| 20b | Cohort characteristics | Partial | Results 3.1; Table 1; Methods Recorded demographics and instrument measures reported; treatments and detailed cancer status unavailable; demographic subgroup descriptions incomplete. |
| 20c | Development/evaluation comparison | Reported | Methods 2.4.3; Results 3.3; Tables S4 and S15 Temporal training/test dates, specimen and unique-patient counts, event counts, and predictor distributions and encoded-data availability are reported. Comparisons are descriptive; clinical variables unavailable in the source remain unavailable. The inherited calendar cut-off is documented; its historical rationale and prespecification status could not be verified, so temporal estimates are exploratory. |
| 21 | Analysis counts | Partial | Results; Supplementary Table S4; code Main, temporal and sensitivity sizes/events reported; per-inner-fold tuning counts are reconstructable from code rather than tabulated in text. |
| 22 | Model availability | Reported | Methods; Supplementary Table S3; code LASSO coefficients, intercept, centering/scaling and executable pipeline supplied. Experimental code is not a clinically approved implementation. |
| 23a | Performance/uncertainty | Partial | Results 3.3–3.6; Figures 2–4; Tables S2, S4–S8, S11 Discrimination, key threshold and Gram coverage/yield intervals, plus calibration-in-the-large/intercept/slope and Brier intervals, are reported. No demographic subgroup metrics are available; bootstrap is conditional on fitted predictions. |
| 23b | Heterogeneity results | Not applicable: not examined | Discussion No cross-center or demographic heterogeneity estimate. Patient-cluster intervals are not such a result. |
| 24 | Updating results | Not applicable: no update | Methods No externally updated/recalibrated model was developed or evaluated. |
| 25 | Interpretation | Partial | Discussion Simple-model comparison, endpoint/coverage caveats and prior literature discussed; explicit fairness interpretation is absent. |
| 26 | Limitations | Reported | Methods; Discussion Selection, sample size, outcome ambiguity/incorporation bias, dependence, conditional uncertainty and external-validation gap addressed. |
| 27a | Input quality | Partial | Discussion; code  Input compatibility and numerical implementation require verification before deployment. No operational failure-handling pathway was validated. |
| 27b | User interaction | Not established | Discussion No deployed interface or tested user-expertise requirements; implementation remains future work. |
| 27c | Future applicability | Reported | Discussion; Conclusion Prospective evaluation, external transportability and clinical impact remain required; routine deferral not supported. |
